# Cultured Meat Safety Research Priorities: Regulatory and Governmental Perspectives

**DOI:** 10.3390/foods12142645

**Published:** 2023-07-08

**Authors:** Kimberly J. Ong, Yadira Tejeda-Saldana, Breanna Duffy, Dwayne Holmes, Kora Kukk, Jo Anne Shatkin

**Affiliations:** 1 Vireo Advisors, LLC, Boston, MA 02130, USA; kora@vireoadvisors.com (K.K.); jashatkin@vireoadvisors.com (J.A.S.); 2 New Harvest Canada, Cochrane, AB T4C 0V2, Canada; yadira@new-harvest.org; 3 New Harvest, Sacramento, CA 95811, USA; breanna@new-harvest.org; 4 Stichting New Harvest Netherlands, 1052 Amsterdam, The Netherlands; dwayne@new-harvest.org

**Keywords:** cultured meat, cellular agriculture, food safety, research priorities, regulatory, interviews, testing methods

## Abstract

As with every new technology, safety demonstration is a critical component of bringing products to market and gaining public acceptance for cultured meat and seafood. This manuscript develops research priorities from the findings of a series of interviews and workshops with governmental scientists and regulators from food safety agencies in fifteen jurisdictions globally. The interviews and workshops aimed to identify the key safety questions and priority areas of research. Participants raised questions about which aspects of cultured meat and seafood production are novel, and the implications of the paucity of public information on the topic. Novel parameters and targets may require the development of new analytical methods or adaptation and validation of existing ones, including for a diversity of product types and processes. Participants emphasized that data sharing of these efforts would be valuable, similar to those already developed and used in the food and pharmaceutical fields. Contributions to such databases from the private and public sectors would speed general understanding as well as efforts to make evaluations more efficient. In turn, these resources, combined with transparent risk assessment, will be critical elements of building consumer trust in cultured meat and seafood products.

## 1. Introduction

Advancements in cell culture technology, particularly those used to grow tissue and organs in the field of regenerative medicine, have led to the prospect of producing meat and seafood products grown from animal cells rather than animal slaughter. These products are known as cultured meat and seafood (CM) and hold promise for environmental and ethical benefits by reducing the need for livestock, especially at the levels required in the current system of intensive animal agriculture. Early and heavy investment into CM by the private sector has driven anticipation over when CM products will reach major markets. For that to happen, they have to pass rigorous scientifically based safety assessments by authorities charged with ensuring the safety of new food products, as well as gain consumer confidence. While CM products have already been approved for sale in Singapore [1], and two cultured chicken products have been authorized for sale in the US [2], these do not cover the full scope of processes and products potentially available from this emerging field and only mark the start of such assessments. Currently, regulators take a case-by-case approach to novel food evaluations and accept data generated using a range of methodologies, as long as they are scientifically valid. More safety research would assist regulators across different jurisdictions in their review of CM products, whether for tastings or access to markets. This manuscript highlights key findings from a series of interviews and workshops held with regulatory and governmental scientists, as a basis to identify priorities for research and the development of essential safety methods, data sets, and standards. This study builds on previous efforts with the industry to advance a safety research strategy designed to accelerate the acceptance of commercial CM products with test methods and knowledge in the public domain.

The work described here constitutes Phase 2 of the Cultured Meat and Safety Initiative (CMSI), a joint initiative between New Harvest and Vireo Advisors aiming to address critical technical, methodological, and informational challenges related to evaluating CM safety. By convening diverse stakeholders from industry, governmental scientists, regulators, academic researchers, and consumers, this work is aiming to bring varied perspectives to advance public knowledge and the practice of food safety for CM products by identifying and addressing current data gaps. Research conducted to develop data and methods build the necessary support elements for the emerging ecosystem, which can raise regulatory and consumer confidence, support industry efforts toward commercialization, and improve the evaluation processes of regulatory safety reviews. Before CMSI’s launch, public conversation around safety assessments of CM products was negligible, despite the fact that the commercial success of CM depends on the development of reliable, scientifically validated methods for safety evaluation. Even in the wake of regulatory decisions, there remains little publicly available data to develop consistent assessment approaches and for the identification and evaluation of public health risks [3]. Ultimately, the performance of practical and applicable safety research will require input and collaboration from a wide group of stakeholders [4].

CMSI Phase 1 convened industry representatives to discuss their safety questions and research needs. It provided a space where innovators openly collaborated and shared previously unpublished data about their manufacturing processes to understand how CM is produced and identify potential hazards. Phase 1 included collaboration with over 50 companies working toward commercialization in this space and led to a peer-reviewed publication that serves as a reference point for hazard identification for developers, policymakers, and others [3,5,6,7].

Phase 2, the findings of which are summarized in this manuscript, aimed to gather insight from the scientists and decision-makers within regulatory bodies tasked with performing safety assessments of these novel food products. A series of interviews and workshops were held with governmental scientists and regulators from 15 jurisdictions around the world to identify governmental priorities for the safety methods, data, and research needed for the regulatory review of CM products to reach commercial markets. The interviews and workshops facilitated discussion about priorities for research and development to support risk analysis, management, and communication by all stakeholders. Governmental scientists and regulators across multiple jurisdictions identified common and actionable priorities for methods and research needed for the development of a comprehensive research strategy for the safety of CM products. 

Following the workshops, researchers from New Harvest and Vireo Advisors collated the input from the participants and summarized the current situation based on the published literature, current food regulations, and established industry best practices in the three topic areas that were of greatest concern:

- The need for research to identify and define the hazards in these novel processes so that appropriate controls can be established to prevent or mitigate hazards, and suitable testing is performed;
- The need for new safety testing methods, particularly in the areas of comparative assessment, input risk assessment, and microbiological assessment;
- The value of publicly available safety data to the public, industry, and researchers.

The results and discussion presented here are a summary of the main topics identified by participants as data gaps, potential safety questions, and research priorities. These research priorities, in conjunction with the priorities identified by the industry in Phase 1, will inform the next steps of the CMSI and future research and data collection that could facilitate the commercial adoption of CM through regulatory approval and consumer acceptance.

## 2. Methods

**Figure 1:**
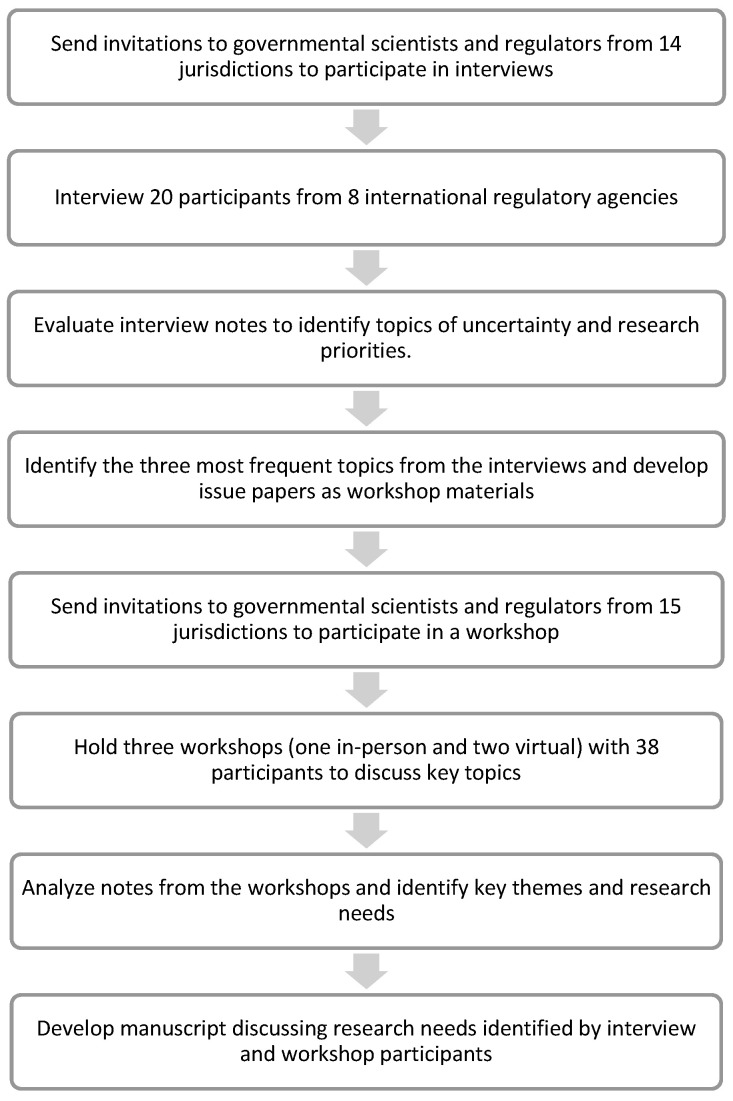
Flow diagram depicting the research methodology.

Sequential efforts were made to identify key research priorities for regulatory review of CM products through interviews and workshop discussions with governmental scientists and regulators from regulatory agencies in diverse jurisdictions (“participants”) (Figure 1). All of the participants are involved in the technical or scientific review of regulatory dossiers for food and are considered qualified to provide relevant information and perspectives on the research needed to demonstrate safety of CM. First, semi-structured interviews were held with participants to identify topics of interest. The interview notes were analyzed to identify key themes for topical discussions during workshops. The topics identified during the interviews and workshops resulted in the set of research priorities discussed here. 

### 2.1. Interviews

Invitations to participate in interviews were sent to governmental scientists and regulators in regulatory agencies from 14 jurisdictions that were known or anticipated to be interested in CM products based on CM company locations, released guidance materials, Memoranda of Understanding, or other public information. Interviews were held with 20 participants from 8 international regulatory agencies. Supplementary Materials S1 provides the list of affiliations of most interview participants. Some participants declined to list their organization for the manuscript, as was allowed per Chatham House Rules. A semi-structured interview guide was used in video conferences with participants, developed around the research priorities identified in CMSI Phase 1 [5]. All interviews lasted 50–60 min and were held under the Chatham House Rule to encourage more open discussion. Chatham House Rule involves an understanding that participants were free to use the information received, but neither the identity nor affiliation of the speakers nor that of any other participant may be revealed. Therefore, data were anonymized and aggregated; identities and affiliations of those who made specific comments are not revealed. 

### 2.2. Post-Interview Analysis

The interview notes were evaluated to identify topics of uncertainty and research priorities for further discussion in the workshops. The topics were analyzed for frequency by counting the number of interviews in which the topic was mentioned by the interviewee (if a topic was brought up multiple times in an interview, it was only counted once). Similar topics were combined as appropriate. 

### 2.3. Workshops 

Invitations to participate in workshops were sent to governmental scientists and regulators from 15 jurisdictions. The overall goal of the workshops was to refine the topics from the interviews to a set of priority research questions reflecting the views of participants. The workshops centered around the top three topics identified during the post-interview analysis (Table 1). Issue papers on the three topics were developed, including background information on the topic and a list of questions raised in interviews (Supplementary Materials S2). Participants were provided with the issue papers in advance of the workshop.

Three separate workshops were held that followed a similar process. With the support of the Singapore Food Agency, an in-person workshop was held at the National Center for Food Science in Singapore on 31 October 2022. To allow for participation from regulatory and governmental scientists that could not attend the in-person workshop, two virtual workshops were held to accommodate participants in different time zones, on 16 and 17 November 2022. There were a total of 38 participants from 13 jurisdictions across the 3 workshops. The list of affiliations of most workshop participants is provided in Supplementary Materials S1. Some participants declined to list their organization for the manuscript, as was allowed per Chatham House Rules. Workshop discussions were organized based on the World Cafe method, a structured conversational process intended to facilitate open and intimate discussion [8]. It links ideas within a larger group to access the “collective intelligence” of the participants and to understand and learn from multiple points of view. Workshops were also held under the Chatham House Rule: data were anonymized and aggregated; identities and affiliations of those who made specific comments are not revealed. 

Workshops started with preliminary information about the goals of the workshop, the World Cafe method, and the 3 discussion topics. Participants were then split into discussion groups (4–8 participants per group) on 1 of the 3 discussion topics. Each breakout group was facilitated by one of our co-authors, and the facilitator for each topic was consistent across all rounds of the 3 workshops. In the discussion groups, participants were asked to consider the types of research and data that would make the safety assessment, hazard identification, and risk management process more reliable and efficient to support the manufacture of safer products. The focus was to explore and innovate on themes rather than problem-solve. At the end of each round, participants moved to a new discussion topic. At the start of each discussion round, the facilitator briefly summarized the previous conversation to motivate further discourse and aim toward convergence on key topics.

### 2.4. Post-Workshop Analysis

The information collected from the three workshops was analyzed and summarized to identify common topics brought up by the participants. While the breakout groups were organized around the themes and questions in the issue papers, the discussions emerged on a variety of topic areas. The workshop notes were analyzed to identify key themes and research needs, independent of the discussion topics. In the results/discussion section, author perspectives on identified research priorities are highlighted with text *in italics*. 

## 3. Results/Discussion

**Figure 2:**
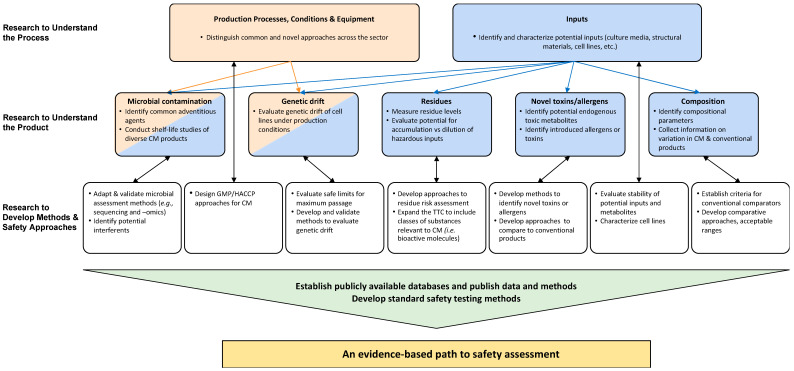
Research priorities identified by governmental scientists and researchers.

Very few agencies have experience in processing full applications for CM products, however, expressed confidence that existing frameworks are applicable to the scope of CM applications. *A recent publication by the FAO outlined existing regulatory frameworks for novel foods across jurisdictions and how they may relate to CM products* [7]*. Most jurisdictions do not have CM products on the market or regulations that specifically address CM food safety, but most do have existing safety approaches that are applicable to CM* [7]. Consequently, the responses to discussion questions were often in the nature of, “we will respond to what we see in the data sets submitted” and “there are too many variables across product types to make general statements of hazard or risk”. It was suggested that an inventory of pre-market food, feed, and environmental safety regulations and policies that may apply in different jurisdictions may be a valuable addition. However, most participants stated that methods were available to evaluate the safety of CM products. It was also indicated that due to the variety of CM product types and processing approaches, not all identified hazards would apply to all CM products. *Therefore, the list of topics for research that CMSI presents in this manuscript should not be viewed as a list of hazards that regulatory experts see for all or even for any individual CM product. Rather, these results aim to identify common and actionable priorities for research and methods needed to develop a comprehensive safety research strategy that reflects the views of governmental scientists and regulators. The research priorities are related to advancing our understanding of the process and its inputs, developing data to understand the products, and developing appropriate analytical methods to measure relevant parameters and perform evidence-based safety assessments (Figure 2).*

### 3.1. Understanding Process, Input, and Product Hazards—Research Needs

The interview and workshop participants identified areas of potential research to better define the process hazards so that appropriate controls can be established to prevent or mitigate hazards, supported by suitable testing to detect potential hazards. The main themes were identified during interviews and analyzed for their frequency (Table 2), along with examples of participant comments.

#### 3.1.1. Defining and Understanding the Production Process, Conditions and Equipment 

Defining and understanding the production process was frequently discussed by participants. Some agreed that although safety requirements might not differ from any other food production process, some aspects may need to be adapted if there are any unique characteristics of the novel process. *Distinguishing the common and novel manufacturing approaches across CM manufacturing could support more efficient safety analysis and the development of guidance or standards. Different stages of the CM manufacturing process align with manufacturing processes from other sectors that could serve as comparators. For example, biopsies or tissue samples are often taken from livestock animals already being raised for food* [9]*. The equipment used for biomass growth and differentiation is similar to those used in precision fermentation and mycoprotein production, although the cell types and media used will differ* [10]*. Finally, the methods used in post-processing of the cell mass are similar to those employed for other processed foods, including the addition of food-grade additives and safe food handling* [11]. Identifying areas of convergence, where certain parts across many CM manufacturing processes could be standardized or generalized, would support more efficient safety evaluation.

Participants identified some areas of convergence across CM production processes; most processes will likely employ bioreactors, standard cell culturing approaches, and similar basal media components*. Identifying the media components that are common across all platforms, such as amino acids, sugars, vitamins, and salts, would support more efficient evaluation and provide more confidence in establishing “safe” levels (e.g., by establishing maximum use or residue limits). Research toward understanding the common adventitious agents or hazardous substances (e.g., cleaning agents) that can be introduced in a bioreactor or during cell culture could support the development of microbiological and toxicological testing guidance or standards.* An example highlighted during the workshops was determining whether antibiotics could pose a food safety risk, similar to use in rearing livestock. *For CM, antibiotics are often used during cell-line development, after which any residue is likely to dilute to safe levels. However, their use further along the process has two-fold implications: increased potential for antibiotic residues above safe levels in the final product, and an indicator of inappropriate or lack of rigorous process controls to manage microbial contaminants. Establishing guidance on the safe use of antibiotics would support the safety of CM products.*

*It* was also recognized that there will be some new manufacturing approaches*. Some novel aspects of production may include the implementation of new techniques to extend cell life, modification of the nutritional quality or sensorial properties such as flavor, color, or texture of meat* [12]*, use of equipment at a scale or designs never used before for food production (e.g., novel bioreactors)* [13]*, as well as introduction of adventitious agents through the cell culturing process that are not commonly found in foods. These novel aspects will require identification and evaluation. It is also important to consider that scaled-up mass production has not yet occurred; therefore, there might be other uncertainties or process conditions that have yet to be considered.*

During the workshops, it was noted that microbiological or chemical contaminants not yet seen in food might be introduced, accumulate, or formed during the production or packaging processes. *Adventitious agents not common to current food processes may be introduced by inputs such as animal-origin media components, handling processes or through environmental contamination. These introduced adventitious agents may potentially accumulate in equipment, such as centrifuges and bioreactors. As the industry continues to develop and adapt equipment for CM production, cleaning and sterilization procedures will require assessment and validation. For example, recycling media could be introduced to reduce the cost of production* [14]*. However, this process may concentrate on certain inputs or byproducts. For example, antinutrients, bioactive substances, vitamins, minerals, and waste may accumulate and change the composition of the final product or become hazardous compounds. Recycling would use separation and filtration methods to remove metabolic waste products and collect non-metabolized inputs for reuse* [7,14]*. The use of sensors to detect and control nutrient feeds will be necessary to make media recycling feasible and to monitor levels of hazardous compounds* [14,15]*. Therefore, it would be beneficial to develop agreed process and product criteria to determine acceptable approaches to mitigating and reducing potential food hazards. A thorough evaluation of Good Manufacturing Practices (GMP) and Hazard and Critical Control Point (HACCP) as suitable systems for ensuring the safety of CM products would support consistent application across the industry.*

#### 3.1.2. Identification and Characterization of Inputs and Residue Levels

*Many inputs such as media, scaffold or microcarriers, antibiotics, and cryoprotectants are required to manufacture CM. Guidelines for assessing the safety of these inputs and their presence in the final product have not been firmly established and are still in development by regulatory agencies* [7]. An overarching theme of the discussions centered around the need to identify all possible inputs and, for each substance, generate data on safe limits and levels found in the final product. Due to the variety of production processes used, this research will need to be performed for different processes. However, an understanding of where there are similarities and differences could inform the level of granularity needed for regulatory review. *For example, some producers may use integral scaffold materials, which are intended to be present in the final product. These materials will have to be established as additives safe for consumption. Other producers may use non-integral structural materials that are removed during harvest and may be considered processing aids* [16]*. Because they are intended to be removed, non-integral structural materials may be more likely to be manufactured from materials not demonstrated to be safe for consumption, which may require residue analysis.*

It will be critical to identify exposure routes and establish safe limits for every input. While some inputs will have a history of safe use in food, inputs that are novel may require a full risk assessment. *Substances that have not historically been used as food processing aids or additives may include bioactive molecules, binding agents, media additives, animal-derived components, or novel scaffold materials. For example, some materials used in tissue engineering historically are approved for clinical applications (e.g., PEG, PCL, PLGA, GELMA) but lack data to show they would be safe to consume in large quantities* [17]*. In addition, food-safe materials may be engineered to improve their use in tissue engineering, such as methacrylation to improve gelation (e.g., κ-carrageenan* [18]*, alginate* [19,20]*, gelatin* [21]) *or addition of cell adhesion moieties* [17,22]*. However, these materials may require a more extensive safety evaluation due to the novel modification* [17]*. Animal-derived components, such as collagen or growth factors, may be present in conventional meat, but their use as a processing aid or food additive may be novel. As another example, phenol red (phenolphthalein) is a media additive commonly used in cell culture as a pH indicator. However, phenolphthalein is not an authorized food additive, may cause allergic reactions, and has been identified as a potential human carcinogen* [23,24]. 

In addition, participants suggested that research is needed to understand the circumstances under which substances accumulate in the product rather than dilute to low levels during the production process. *The use of some substances early in the production process, such as cryoprotectants, relies on the assumption that their final product concentration will be diluted to a level below hazardous thresholds due to large fluid exchanges that occur during production. However, some substances may accumulate if they are sequestered onto structural materials or internalized but not metabolized by cells, leading to accumulation. In some cases, this could be an intentional component of the process, such as the sequestration of growth factors by structural materials to amplify their signals and reduce media costs* [25,26]. Participants also identified medium recycling as another process that may cause inputs or metabolites to accumulate [7,14].

The participants noted that many of these inputs do not have existing “food grade” standards. *As the industry moves towards increasing production levels and decreasing production costs compared to their use in pharmaceutical production, it will be important to establish safe levels as well as industry standards and specifications for quality and purity* [14]. However, it was noted that “food grade” is an industry-standard, not a regulatory standard. 

Overall, because of the variety of inputs and production processes used across the industry, participants recognized that regulatory review of inputs will need to be performed on a case-by-case basis, at least in the near term. Participants expressed an understanding that standardizing inputs too early may limit innovation in the field. However, the collection and open sharing of data could guide the development of standards and specifications for inputs, which are required for the creation of consistent, streamlined regulatory review processes. In addition, participants recognized that transparent risk assessment for all inputs is critical for consumer trust.

**Bioactive molecules**

Bioactive molecules, which have the capability to interact with components of living tissue to produce a physiological effect, were a specific area of concern. *While many bioactive compounds are present in conventional foods, some bioactive molecules are expected to be new to food production or used in novel ways for the production of CM, such as small molecules, hormones, or growth factors* [27]. Participants noted that while many are naturally present in conventional meat and seafood, the majority of these inputs have no previous use as additives in food production and therefore do not have available toxicology information or existing risk assessments. Particular concern arose from the potential for recombinant proteins that have similarities with those present in humans, either because human recombinant proteins are used *or due to similarities with other mammalian species* [28,29] *which are used as pharmacologically active substances. For example, bovine IGF-1 is known to be structurally identical to human IGF-1; in the 1990s the Joint FAO/WHO Expert Committee on Food Additives (JECFA) concluded that it is likely to have a similar effect in humans* [30]. For many bioactive molecules, there is a lack of research on their use in food and their potential consequences, such as potential autoimmune effects [31]. *Some research shows the potential for certain proteins to trigger an autoimmune response after consumption by sensitive populations, such as infants* [32]*. Other studies have shown autoimmune responses due to the use of certain proteins in clinical procedures* [33]*. However, limited information is available on the potential for the substances used in CM to induce an autoimmune response after consumption.* Previous work on growth factors and their use as veterinary or clinical drugs could be a useful starting point for research on their use in CM. *Extensive research has been performed on the bioactivity of bovine IGF-1 following oral intake due to concerns surrounding the use of recombinant bovine somatotrophins (rBSTs) in dairy cows* [30,34]*. Research to establish methods that assess the safety of bioactive molecules would address these concerns.*

**Cells**

Considering cells themselves as an input, participants considered the variety of cell types and manipulations that could be used in CM production. *Cells for CM production may be primary cells taken from animal biopsies or cells derived from established pluripotent or multipotent cell lines* [17]*. While significant research has been conducted on primary cells, these cells typically exhibit limitations in population doublings resulting in cell senescence and the need for repeated cell sourcing, making primary cells undesirable for large-scale CM production* [35,36]*. For this reason, the development of cell lines capable of extended proliferation, whether pluripotent stem cells (ESC, iPSC) or “immortalized” cells (through induced or spontaneous mutations), is desirable* [37]. Participants discussed uncontrolled cell proliferation and related concepts of tumorigenicity. However, they did not indicate concerns directly related to the tumorigenicity of ingested cells and rather raised this concern in relation to risk communication and public perception. *The FAO and WHO in their recent expert consultation on food safety aspects of cell-based food addressed this concern as out of scope for hazard identification* [7]*. Experts concluded that the sequence of events necessary for a pluripotent or immortalized cell to survive and form tumors after consumption was unlikely and not consistent with current scientific understanding* [7]. Although experts are unable to identify a credible pathway to harm, participants acknowledged that careful risk communication or testing to resolve any scientific questions about tumorigenicity may be helpful for addressing consumer perceptions related to immortalized cells. 

#### 3.1.3. Stability of Inputs and Metabolites

Participants noted that research is needed to characterize and quantify the stability of inputs and the potential metabolites produced during production, during storage, or following consumption. Metabolites may be formed due to the breakdown of the inputs or cross-reactivity with other substances. In addition, participants noted that after consumption, interaction with the gut microbiome may further metabolize input substances, adding another level of complexity. *Extensive work on the formation of metabolites has been performed in the pharmaceutical industry, where drug metabolite toxicity must be evaluated* [38,39] *and may serve as a resource*. For some novel inputs (e.g., polymer scaffolds), there is limited information about potential metabolites or degradation products and any associated toxicity or allergenicity [22]. Even in the case of inputs present in conventional agriculture, their stability and metabolite formation may be different under cell culture conditions. Studies on compounds in conventional meat are a good starting point, however, further research is needed to validate the findings in CM manufacturing processes. *An understanding of input stability will allow for standards and specifications related to culture conditions, shelf life, and cooking recommendations, limiting the potential for harmful metabolite formation.*

Specific to bioactive molecules, research is needed to assess whether the inputs remain bioactive in the final products and after consumption. *Due to the heat instability of many bioactive molecules, processing or cooking the CM products may inactivate these substances (though not all products are served cooked)* [40,41]*. Bioactive molecules may also be rendered inert during digestion* [42]. However, the inactivation of bioactive compounds after cooking and digestion requires research and validation.

**Cell stability**

Participants also discussed a need to study the genomic and metabolomic stability of cells (i.e., genetic drift) with prolonged passage and possible changes in expression products that could be hazardous or allergenic. *Establishing cell lines capable of long-term culture is highly desirable for CM producers to limit batch-to-batch variability. However, shifts in the genetic and phenotypic properties of the cells can occur as a result of physical or biochemical stimuli during culture, extended cell division, or mycoplasma contamination (among others). In addition, extended culture time can select for cells with altered growth characteristics* [43]. Methods may be needed to detect changes in cell stability when there is a deviation from standard operating protocols. Participants noted that changes in the expression products of cells could lead to new metabolites. Research is needed to establish approaches to set safe limits for maximum passage and to establish appropriate parameters to monitor and characterize stability. Participants highlighted that an open-access database could be useful to generate large datasets to track genetic drift in CM-relevant cell lines and support the safe use of cell lines. Knowledge of which parameters to evaluate during cell culturing would be valuable.

#### 3.1.4. Novel Toxins/Allergens

Participants noted there is a need to assess the capability of animal cells to endogenously produce hazardous substances, such as toxins or allergens. *The majority of endogenous biological toxins are produced by bacteria, fungi, algae, and plants, rather than the mammalian, avian, or seafood species that will be used for CM* [44]*. However, biological toxins can be harmful in very small quantities;* therefore, participants expressed a need to characterize cell lines and identify potential expressions of toxins or allergens. *There is a range of species that may be used for CM production that have the potential to produce toxins. Certain fish, snakes, jellyfish, and toads, along with a few mammalian species, such as some shrews, the platypus, vampire bats, and slow and pygmy lorises, are capable of producing venoms* [45,46]*. Studies demonstrate that it is possible to culture toxin-producing cells* in vitro [47]*, though it is unknown if the conditions to produce CM would support expression of these proteins. Databases such as the animal toxin annotation project (UniProt) and the Animal Toxin Database (ATBD) may serve as tools to identify potential proteins secreted in animal venom. There may not be data available for species with a limited history of consumption, for which the potential to produce novel toxins or allergens may require further evaluation.*

While there were no food-related toxins of concern that are produced by animal cell lines identified during the workshop, it was suggested that work from areas such as human cancer research could be mined to identify potential endogenous toxic metabolites. *Cells have the potential to produce endogenous toxic metabolites that, if consumed chronically, may contribute to adverse health outcomes. For example, metabolites that contain reactive groups such as methylglyoxal, 4-hydroxynonenal, and glutaconyl-CoA may accumulate* [48]*. However, whether these metabolites could pose a food safety hazard is unknown. Accumulation of these substances in cells is likely to kill the cells in vitro, thereby reducing the ability to enter the food stream. Research on whether low levels of these metabolites may pose a long-term food safety risk may be needed.*

### 3.2. Developing Analytical Methods and Safety Approaches—Research Needs

The interviewees and workshop participants identified a number of areas of potential research in developing new safety testing methods, particularly in the areas of comparative assessment, input risk assessment, and microbiological assessment.

#### 3.2.1. Testing Methods—Comparative Assessment

One of the key topics of discussion was whether compositional analysis and comparison to conventional meat were useful as a tool for safety assessment. Development of an appropriate approach to evaluate the similarity or identify compositional differences between CM to conventional products based on final product analyses may require further development.

**Comparative approach**

The ability and need to establish a comparator was discussed. Some highlighted that there may not be appropriate conventional comparators for some CM products (e.g., a conventional piece of meat). Some parameters such as protein content, amino acid ratios, and materials used in scaffolding are expected to be different from conventional meat; these may not necessarily be a safety issue. *Choosing an appropriate comparator and understanding the similarity of CM to conventional products may help develop dietary exposure evaluations, using data on existing intakes of products of similar nutritional composition.*

It was highlighted that conventional meat already has a wide range of natural variation; collecting this information would be valuable for comparative purposes, as it is unclear what range of variation in CM could be considered safe and nutritious. *Meat quality and composition can vary based on animal genetics, rearing conditions, and the nutritional status of the animal* [49]*. Similarly, CM properties may vary based on the genetics of the source animal, modifications to the cells, and culture conditions. Research on the range of variability and linking to acceptability in terms of nutrition and human health effects would support the identification of appropriate comparators.* It can be envisioned that in the future, CM may be produced from animals that are not conventionally eaten, such as extinct or endangered species. *In these cases, research will be required to determine appropriate comparators or parameters to evaluate safety, as it could be considered unethical or impossible to take samples from their conventional counterparts.*

Regarding the types of comparative parameters that could be used to support safety and/or nutritional assessment, some participants suggested drawing from historical approaches. These include parameters used for the evaluation of conventional food and food additives, genetically modified (GM) plants and GM animals for food, and drugs as starting points. *For example, the evaluation of genetically engineered animals intended for food, such as the AquAdvantage salmon and GalSafe pig, determined potential adverse effects from consumption by measuring the proximate, vitamin, mineral, amino acid, and fatty acid parameters of the edible tissue along with assessing the growth hormones present in tissue* [50,51,52].

**Nutritional evaluations**

Participants noted that many of the compositional parameters may support nutritional assessment but not necessarily impact food safety. *If CM were to be a complete substitute for conventional meat sources, an understanding of how different ratios of various components can affect nutritional balance is an important research question. Long-term consumption of CM as a substitute for other sources of protein may lead to adverse nutritional outcomes, such as vitamin deficiency or excess intake.* There was recognition that some products may be fortified (e.g., the addition of vitamins); there is already experience in evaluating other types of fortified products [53], which may be applied to CM. However, more research may be needed in this area specific to CM. 

*Generally, amino acid, mineral, and vitamin intake from dietary sources do not contribute to toxicity or poisoning* [54]*. Conventional meat and seafood are valuable sources of vitamin B12, iron, zinc, selenium, phosphorus, essential amino acids, and certain fatty acids* [55,56]*. Additionally, animal protein is considered a good dietary source of amino acids, contributing to a healthy human diet* [57]*. Research is needed to determine the differences in the composition of CM products compared to conventional counterparts which could create long-term nutritional imbalances in consumers. A better understanding of how to analyze and interpret results is also needed; while some measured variables may be statistically significant, the biological relevance (i.e., meaningful or actual adverse outcomes in humans as a result of the change) is not well defined and requires specific consideration* [58]*. Research may also be needed to evaluate whether these parameters capture nutritional quality. Assessing whether CM products provide similar protein quality, digestibility and nutrient availability to conventional meats (e.g., with Protein Digestibility-Corrected Amino Acid Scores [PDCAAS]) is a needed area of research and may require longer term studies* [56].

It was also emphasized that from a nutritional perspective, many foods we eat (including meat) are not necessarily “healthy” (e.g., high in saturated fats), so some comparative parameters may not be of value. *It may not be desirable to produce CM products that have the same nutritional profile as its conventional comparator. For example, there is an opportunity to reduce unhealthy saturated fats or increase vitamins in CM* [9,59]*. In these cases, a direct comparison to conventional products may not be appropriate. Instead, an analysis of these parameters can be conducted independently of a conventional comparator.* In addition, it is anticipated that many CM products (at least initially), would be combined with other ingredients; in these cases, comparators to processed foods may be more appropriate than conventional meat.

Participants noted that the development of specifications or standards of identity may be a useful area of research. *Development of specifications, such as the Codex General Standard for Food Additives* [60]*, or standards of identity (such as ones developed by regulatory agencies for meat and other food additives) could be useful in identifying consistent CM safety and quality parameters, such as gross composition and mandatory versus optional ingredients.*

#### 3.2.2. Testing Methods—Safety Assessment of Inputs

There was general agreement that an analysis of the potentially harmful contaminants, residues, and metabolites of inputs would support safety assessments. Once an understanding of the possible inputs is obtained, research is needed to identify effective quantitative testing methods. While many toxicity and allergenicity tests currently used in food or pharmaceuticals could be applicable, they may need to be adapted and validated for new targets or for novel matrices (e.g., cells and scaffold with media). 

Participants discussed that it will be important to determine whether testing should be conducted on whole products or individual substances. While it will be important to understand how the body deals with the product as a whole, testing on individual substances may be needed to establish toxicological data on safe limits for substances not found in conventional foods. While the current standard is animal testing, some participants expressed concern about using this approach for whole foods, in which identifying the specific compound causing any adverse effects is challenging. For many substances, animals would need to be fed for extended periods of time to achieve relevant measure outcomes. In addition, there are concerns about using animal testing for products intended to reduce the use of animals. *In recent years, alternatives to animal testing for food safety assessments have been under investigation. Alternatives may be employed such as read-across, high-throughput screening,* in vitro *testing,* in silico *models, organoids, and organ-on-a-chip technologies* [61,62]. 

It was suggested that there are likely to be different approaches to evaluating the safety of endogenous vs exogenous substances. A comparative approach may be more appropriate for endogenous substances (e.g., amino acid), and a more traditional safety assessment (e.g., hazard and exposure evaluation, use of a threshold of toxicological concern (TTC) concept) appropriate for exogenous substances (e.g., cryoprotectants). For endogenous substances, an identical or similar substance may already be found in meat products (e.g., growth factors), for which the existing literature can be used to determine a safe threshold for consumption. *IGF-1, discussed above, is an example of an endogenous substance with extensive research in conventional animal foods which can be applied to its use as an input for CM production.* For exogenous substances, further research is likely necessary to establish safety. Some substances, such as antibiotics, may already have established use in meat production and, therefore, existing toxicology data [63]. *In these cases, screening methods for the final product may be straightforward. However, many exogenous substances will not have a precedent for use in food. For example, the small molecule, SB 203580, has been suggested to maintain bovine satellite cell stemness for large-scale CM production* [64]. For establishing safe limits, traditional hazard and exposure evaluation may be appropriate. A relevant concept, which participants repeatedly mentioned, is the Threshold of Toxicological Concern (TTC). *TTC is a methodology used to assess the safety of food substances of unknown toxicity when the chemical structure of the substance is known, and the estimated exposure is low* [65]. For chemical contaminants, using the TTC may be useful and an opportunity for implementing a “read-across” approach [66]. However, some substances might not have enough information, and further analysis may be warranted if areas of concern are identified. Overall, the focus should be on hazards that are relevant to human health, and thus, food safety. However, participants expressed concern that the application of the TTC may not be sufficient for all CM inputs, especially for bioactive molecules. Additional research is required to extend the TTC methodology to include these classes of substances, including criteria for the stability of proteins.

*Regarding allergenicity, as for all foods, caution needs to be taken to avoid introducing known allergens* [67,68]*. Existing allergen detection methods such as immunoassays can be applied to identify known allergens* [69]*. Theoretically, any novel protein could be allergenic. However, there is no well-defined assessment of allergenicity* [68]*. To assess the potential allergenicity of novel proteins, the current approach is a “weight-of-evidence” approach, where proteins can be screened for similarity to known allergens and evaluated on the basis of molecular weight and stability* [70]*. One challenge may be to identify novel proteins that cause de novo sensitization* [70]*. Therefore, it may be important to assess the potential allergenicity hazard for novel proteins in the final product.*

**Input Stability**

Participants suggested a few different ways that input stability could be studied for their assessment in CM products. In vitro and in silico experiments can be used to assess relative measures of stability. Studies using selective pressures on classes of inputs would be beneficial to predict the full range of potential breakdown products. *For proteins, work can be taken from the pharmaceutical industry, in which understanding the stability and metabolites of small molecules and therapeutic peptides and proteins (TPPs) is critical. Several biophysical methods and high-throughput screening approaches exist to measure protein stability* [71]. In addition, proteomics and metabolomics techniques can be used to explore the full possibility space of potential metabolites. *Recently, top-down differential mass spectrometry was shown to be an efficient method of discovering protein metabolites for TPPs* [72]*. Similar work can be conducted using metabolomics for other small molecules as well.* In vivo studies could be used to understand potential metabolites formed after consumption. However, participants suggested these studies are difficult and highly variable. *Some* in vivo *conditions can be assessed with biochemical or* in vitro *high throughput studies, evaluating effects such as temperature, pH, and enzymatic activity* [71]*. Cutting-edge work aims to develop organ-on-a-chip models of the human intestine that could replace animal models* [73]*. Additionally, studies that evaluate the effects of substances on the microbiome might answer some questions, but standardized methods are not currently available.*

**Cell Stability**

It was also discussed that screening will be important to identify mutations or changes in the cells (i.e., genetic drift or genetic modification) that could result in hazardous metabolites or allergens. Screening could be conducted at the genomic, proteomic, or metabolomic levels. Approaches for evaluating genetic drift may be taken from the pharmaceutical field, though methods are not well-established or standardized for food production. *A large-scale study of 1497 cell lines across the three largest pharmacogenomic studies found extensive genetic drift* [74]*. Karyotyping can be used to analyze the total chromosomal content of individual cells, identifying chromosomal changes from aneuploidy, which is common in established cell lines, to small deletions, duplications, inventions, or translations* [43]*. Proteomic and metabolomic analysis can also be used to monitor changes in expression or metabolites.* There was recognition that identifying metabolites, in particular, would be a challenge as metabolomic analyses generate large, hard-to-analyze datasets. Research using hypothesis-driven analyses could help focus on actual hazards; *however, biological pathways leading to metabolites of concern must first be identified in order to perform targeted metabolomics*. Comparative metabolomics could help define the similarities and differences in the starting material as well as conventional products, and approaches such as principal component analysis (PCA) may help to “group” products. 

#### 3.2.3. Testing Methods—Microbiological

During the workshops, several participants mentioned the importance of determining if current monitoring, sampling methods, and frequencies are appropriate or need to be adapted for assessing the safety of CM products. Some participants suggested that there is a plethora of traditional and high-tech assessment methods that have been designed for existing hazards. *Although traditional culture-based detection methods are still the gold standard, they are being substituted by faster and more sensitive alternatives such as immunological, molecular, or spectroscopic methods* [75,76]. One example that was brought up during discussions was metagenomics*. Applying next-generation sequencing (NGS) to identify genetic sequences of multiple microorganisms in a sample is relatively new in the food safety sector. Nevertheless, metagenomics could provide an opportunity to detect, identify and characterize the microbiota or a broad selection of pathogens, if any, in CM products* [75,77]*. Metagenomics can be used as a routine monitoring tool for raw materials and final products to help identify emerging or unknown hazards (e.g., bacteria, viruses), which could be invaluable for novel cultured food products* [78]*. In addition to metagenomics, other novel tools that could be explored to assess the microbial food safety of CM products are transcriptomics, metabolomics, and proteomics, which are commonly referred to as “foodomics”* [75,78]. 

However, regardless of the variety of available methods, participants were aware the selected tools would need to be suitable for assessing CM products. *Several elements would need to be considered when choosing a detection method such as the target to be identified, the microbial load, the speed of detection, the sensitivity of the method, and the food matrix* [75]. Participants agreed the influence of the novel food matrices of CM will need to be considered when choosing microbial detection methods. Current available methods are validated for conventional food products; they might need to be validated or recalibrated for new food matrices or potential new hazards. *Food composition can affect the performance of detection methods; thus, it is common that complex food samples undergo a preanalytical preparation that can eliminate interfering substances or concentrate and purify the target pathogen* [75]*. The latter is more relevant for sensitive molecular methods* [75]*. For example, two ingredients that could be used in CM, gelatin and fat, can interfere with PCR and reduce diagnostic sensitivity and specificity* [79,80]*. Certain quantities of sucrose, sodium chloride, and lysine have also been shown to inhibit PCR* [81]*. In addition, chemical and physical properties such as pH, density, and adsorption of components, have also been shown to interfere with the performance of other types of detection methods such as surface plasmon resonance (SPR)* [81]. The more complex the food matrix, the more complex the pretreatment and assessment might be. *That is the case for raw meat products. Samples of ground beef and chicken normally are blended or homogenized before they are assessed, which could release antimicrobial components or enzymes with the potential to interfere with detection methods* [81]*. Another potential barrier to microbiological evaluation is the sample size. For example, many standard pathogen detection methods require an enrichment step that is selective to recover a specific pathogen in a relatively large sample (e.g., 25 g). Access to large samples is currently a barrier to assessing CM, thus methods might need to be adjusted.*

Another concern brought up by participants was that the food matrix composition not only could interfere with detection methods but could also affect the microbial growth of known pathogens or emerging ones. *Pathogens in food can be found in low concentrations and or sublethally injured and heterogeneously distributed within the food matrix, increasing the challenge of detection* [81]*. Therefore, sample pooling, isolation, and concentration techniques have become a common practice to aid detection and avoid growth inhibition from food matrix components particularly when an enrichment step is necessary* [75,81]*. Due to the novelty and highly industry-driven nature of CM products, public information regarding their composition is lacking. However, the available literature suggests it could be highly variable* [9,80]*. For example, it is possible that products could contain additional structure-forming ingredients such as hydrocolloids, starches, or fibers to help with the gelling and emulsifying properties cultured cells may lack* [12]*. Therefore, for microbial assessment, there is a need for research to properly verify and validate the methods used for the detection of microbial contaminants in a particular cultured food matrix.*

Participants acknowledge that although CM could be less susceptible to contamination than their conventional counterparts, contamination is still plausible throughout the manufacturing process. *Evaluation of the potential for microbiological contamination from the source animal or cells, such as bacteria, viruses, and prions may be the first step in qualifying cells for CM production* [5]*. Cell culturing needs to be carried out in aseptic conditions, which reduces the risk of contamination* [80,82]*. However, aseptic conditions may be challenging to maintain after the cells are harvested and they are ready to be processed into a food product, especially once the manufacturing process is scaled-up* [5,82]. Although “sterile” has often been used to describe the final product and process, some participants agreed that it might not be feasible (nor desirable) to mass produce a “sterile” final food product. *The major limitation may be cost, as one study assessing the economic viability of CM highlighted the likelihood of high capital costs of equipment and facilities to ensure sterility* [83]*. Though bulk cell production (i.e., in bioreactors) might be desirable to occur under similar sterile conditions as the biopharma industry, it is likely that facilities could follow less stringent conditions because the food processing stage would occur under similar conditions to other conventional food processes* [83]. Participants pointed out that background microflora could be different from the one found in conventional meat, or it might be lacking, allowing pathogens to grow easily. Evaluating the implications of processing, storing, and transporting a sterile product is identified as a research need. In addition, the native microflora can affect the reliability of some standard tests. *Studies have shown that during food microbial assessment, native food microflora can grow and compete with pathogens hindering their growth and further detection* [84,85]*, particularly in raw food* [81]*. For example, a study found that the growth of L. monocytogenes in different enrichment broths is highly dependent on the composition and initial numbers of the native food microflora in combination with the ability of the selective media to inhibit the growth of the competitors* [84]*. Therefore, the presence or absence of native or transitory microflora in CM products will need to be assessed. Establishing real-time monitoring protocols with the help of sensors at critical stages could help detect microbial contamination before it spreads* [80,86]*. However, the monitoring and control points will be dependent on the scale of production* [87]*. Furthermore, it may be relevant to identify the most common pathogens in CM production and to better understand their behavior. This will help properly identify the optimal detection methods, develop predictive microbiological models, and establish the proper monitoring and sampling programs adequate for the unique manufacturing process conditions* [84,85]*. Currently, the lack of access to CM samples and process data has prevented public research on microbial assessment to be conducted. Therefore, there is still a gap in data where more open collaboration between industry and academia could be beneficial.*

*Understanding the food matrix composition, physical and chemical properties, and potential presence or absence of microflora will play a key role in determining the type of packaging technologies that can be used and/or the stability of the final product* [88]. The latter was also emphasized during the workshops when participants brought up the need to perform shelf-life studies on the final product. *It is still unknown if CM products will have an extended shelf life when compared to their conventional counterparts. Detailed profiles of the product’s shelf life may need to be developed. For example, for meat and fish products, some parameters that are relevant for shelf-life profiles are color, pH, water holding capacity, moisture, and total viable counts, among others* [89]*. Access to diverse products along with more research is needed to assess physicochemical and microbiological activity that could cause spoilage and reduced shelf life* [89]. 

There was also agreement among several participants that the final product would need to be assessed to establish appropriate handling and preparation instructions. An example brought up during the discussions was assessing the presence of retroviruses in uncooked products, such as raw fish, which may be less relevant for cooked products. *Although it is still unknown if retroviral DNA will persist in culture, a study assessing the thermal stability of diverse viruses relevant to food safety concluded that not all viruses were inactivated by common thermal food processing methods* [90]*. Therefore, more research needs to be conducted to assess the potential microbial risks that persist after the preparation of CM products. Additionally, this brings a bigger* question that was also posed during the interviews: will raw CM have the same safety hazards as conventional products and will consumers understand this difference, if any? *To ensure conventional meat and seafood products are safe for consumers, there are established guidelines, standards, and specifications that highlight acceptable microbial limits. However, for CM products, there is a lack of public studies that could be used to establish similar guidelines, standards, and specifications. Therefore, it is still unknown whether CM products will possess similar microbiological hazards as their conventional counterparts.*

### 3.3. Establishing Publicly Available Databases and Information

#### 3.3.1. Databases

Many participants mentioned that established databases are available with comprehensive data including composition (USDA Food Data Central, FOODB), metabolomics (NIH Metabolomics Workbench, The Human Metabolome Database), and proteomics (Uniprot) for food and other relevant products. These include data on genetically modified plants (FAO GM Foods Platform), antibiotic residues (Codex Veterinary Drug Residue in Food Online Database), allergens (Allergen Online), and human disease (Human Cell Atlas, Human Protein Atlas). *However, much of the work in proteomics and metabolomics has centered around medicine, and there is a lack of data on proteins and metabolites found in food products* [91]. It was suggested that the creation of similar databases for CM that aggregate research data and compositional parameters would be valuable for the field as a whole, including regulators, researchers, and the industry. These types of databases would also be valuable for contaminants and residues, such as common media components. *There are some existing databases on serum-free medium formulations, although detailed information on media components is not readily available and is often proprietary (FCS-free Database). Proteomics data are available on fetal bovine serum and may be valuable, although CM developers are moving away from its use in media* [92]. However, participants acknowledge that, historically, this type of data has often been generated reactively due to a crisis, rather than proactively, because there is little funding or incentive for scientists to do this type of data collection early during a technology’s development. *Waiting to develop this data after a crisis occurs risks significantly hindering the progress and adoption of CM. Coordinated efforts from regulators, funders, and scientists could help to develop these data early in CM development rather than retroactively, avoiding safety emergencies and expediting the progress of CM to market.*

Many participants also suggested having these databases be open access to facilitate information sharing, hazard identification, and transparent communication with diverse stakeholders, including the public. However, other participants highlighted the challenges to making this practical and useful, acknowledging that this would need to be conducted in a coordinated way, likely requiring a neutral third party to develop, maintain, and coordinate data collection and secure funding. *Examples of existing databases illustrate how these are typically organized by governmental or intergovernmental organizations, such as the USDA and FAO, often in partnership with nationally funded research institutes. However, the Human Cell Atlas provides an example of collaboration among international researchers. In most cases, a central component to the success of a database is a governing body to coordinate, process, and quality-control the data provided. The Human Genome Project (HGP) is another example illustrating the need for coordinated data sharing. Although the HGP was initially successful in allowing researchers to openly and immediately exchange information, it led to the establishment of diverse databases with different requirements and policies for data sharing that make it challenging for researchers to access data. Therefore, in 2013, the Global Alliance for Genomics and Health (GA4GH) was established to create standards for databases to harmonize data and coordinate efforts to make sharing easier worldwide* [93].

Participants also brought up the challenge of accessing proprietary information from companies, and the need to balance information sharing and the protection of IP. *Innovative technologies such as CM inherently require knowledge exchange and collaboration to advance* [94]*. However, little attention has been given to addressing how to balance the two opposing approaches, knowledge sharing and protection, so companies maintain their competitive advantage* [95]*. The Western Growers GreenLink^TM^ platform started in 2017 and provides an example of data sharing within a consortium of industry members* [96,97]*. Anonymized food safety data are shared within a secure and confidential digital platform. When designing GreenLink, the protection of proprietary information was critical and currently, the database is only accessible to members of the organization. Initial results shared by Western Growers highlight its value to anticipate, predict, and plan for food safety and the importance of industry buy-in and feedback when designing these tools.*

#### 3.3.2. Value of Safety Assessment and Data

Although several unknowns and research gaps were identified, some participants believed that CM products might not be that different from conventional products from a microbiological and toxicological standpoint. Many participants expressed the view that there were likely few novel hazards and that methods to control and test for hazards could be adopted from existing food processes or other similar industries. However, due to the lack of available industrially relevant manufacturing information, published safety studies, and standardization or validation of existing testing approaches for CM safety assessment, there is a lack of transparency and scarcity of publicly available scientific data to support safe consumption of CM. 

The publication of these data is not only important to regulators for safety and nutrition evaluations, but to provide more information to consumers. Several participants expressed the need to appropriately frame the issues so the context for safety assessment can be understood. *Consumers might perceive eating CM as a personal risk due to the uncertainty and fear of the unknown* [9,98]*. Others have also found that concerns about perceived unnaturalness can be linked to concerns about safety* [99,100]*. In fact, unnaturalness has been found to be the main cause of health and safety concerns among consumers* [101]. Therefore, as discussed during the workshop, demonstrating that CM is “similar” to conventional products may provide some assurance of safety, but may also be important in relation to other aspects of consumer acceptance such as taste, nutrition, naturalness, cultural beliefs, etc. *Studies demonstrate that consumers are concerned that CM will not taste good or that is not natural* [80,100]*. However, it will be important that consumers understand that CM could allow the production of healthier meat products, such as reducing saturated fats and increasing unsaturated ones or engineering nutritionally enhanced CM* [59]*. Publication of compositional and safety analyses reviewed by scientists may increase transparency and support consumer acceptance by demonstrating that products are similar to conventional products, and do not contain any hazardous ingredients.*

*Public perception has been brought up before by other experts to understand where potential safety concerns could arise, and how companies need to be transparent with consumers, particularly with labeling* [4]*. In addition, it will be extremely important that proper regulations and process controls are in place to ensure safety and that consumers are aware that proper government oversight is in place* [80]*. Reducing misconceptions and concerns from consumers will require adopting an interdisciplinary approach where CM technology meets behavioral science* [80].

Research is needed to identify credible risks and evaluate potential public health consequences based on commercial manufacturing practices. Participants acknowledged that the lack of public data and details on the manufacturing process creates public and scientific uncertainty. *Currently, hazard identification and risk evaluation are performed on a case-by-case basis, without much public disclosure*. *There are some potential hazards where experts do not believe there is a probable sequence of events that could lead to an adverse outcome. For example, a group of FAO experts evaluated the potential for the consumption of cultured cells to lead to tumorigenicity and concluded that the sequence of events leading to this outcome is highly improbable* [7]*. Credible scientific research to validate these assumptions was seen as a way to address the questions that consumers have. Performing safety research and making results publicly available will be crucial to gaining public trust, but also to support consumer understanding of the products they are consuming*.

## 4. Conclusions

Interviews and workshop discussions with governmental scientists and regulators identified a number of sources of uncertainty as well as priority questions about the safety assessment of CM products. However, while novel elements to production might contain new food safety hazards, it was considered likely that existing approaches for demonstrating food safety can be adapted to assess the safety of CM products.

At the compositional level, CM products may vary widely in terms of similarity with conventional counterparts, creating a need to develop criteria and methods for comparing identity between cultured and traditional products. There is also a need to understand which parameters are relevant for evaluating safety—separate from information useful to consumers (e.g., nutrition)—as well as streamline safety approaches, easing the burden on regulators and the industry.

At the process level, the “how” of growing and converting animal cells into food ingredients or products is where experts expect the greatest differences from traditional products and where novel parameters and analytical methods may have to be developed. The genetic and metabolic stability of cells was raised, specifically how changes over time could generate potential hazards not present in traditional products. There was consensus that testing for the presence and concentration of residues be conducted for all potentially hazardous inputs, such as media ingredients, structural materials, and cells. Bioactive molecules may be of particular concern since they do not have a history of use as an additive or process aid in conventional food production, and there may be a lack of available safety information. Further research on accumulation and interaction with other molecules may be needed for inputs, as well as the efficacy of monitoring or remediation strategies for hazardous substances.

Evaluating and identifying microbiological and chemical contaminants was also of concern. There was wide agreement among participants that common methods of hazard prevention and mitigation (HACCP, GMP) would be sufficient for establishing food safety once sources of contamination are understood. Pathogens and chemical compounds commonly found during traditional meat and seafood production may be relevant to CM production, particularly in downstream environments (e.g., food processing and storage), while upstream manufacturing processes (cell culture) may introduce pathogens or chemical compounds not typically seen in conventional food production. Sterile final products or those with unique microbiota may present benefits as well as challenges regarding contamination, spoilage, and shelf life.

Overall, participants suggested that the identification of novel parameters and targets may require the development of new analytical methods or the substantiation and adaptation of existing approaches to evaluate composition, inputs, and contaminants.

Given the potential variety of processes and products, participants agreed that a common list of expected parameters or analytics is not currently suitable for the safety evaluation of any and all CM products. Rather, for the foreseeable future, they will have to be assessed on a case-by-case basis. Over time there may be some convergence in production processes, product composition, and safety analytics. Research to identify such convergences will be important as any generalization or standardization can lead to more efficient safety evaluation and commercialization.

Toward this end, participants repeatedly expressed the need for more research and data in the public domain to be able to evaluate any new hazards. Public data are needed for a diversity of products and processes, such as the development of databases similar to those already available in the food and pharmaceutical fields. Contributions to such databases from the private and public sectors would improve the identification of hazards common across CM manufacturing and support more efficient safety analysis and development of regulatory frameworks and standards. In turn, these resources, combined with transparent risk assessment, will be critical elements of building consumer trust in CM products.

## 5. Takeaways

Participants identified key research priorities to support evidence-based safety assessment of CM products (Figure 2).

*Research needed to understand the process*

- Distinguishing common and novel manufacturing approaches across the sector;
- Identifying and characterizing possible inputs (e.g., culture media components, structural materials, cell lines, etc.).

*Research needed to understand the product*

- Identifying common adventitious agents that can be introduced during manufacture and be present in the final product;
- Conducting shelf life studies of diverse CM products;
- Studying genetic drift of cell lines under production conditions;
- Establishing data on residue levels and potential metabolites of inputs;
- Evaluating the potential for accumulation versus dilution of chemical or biological contaminants or toxicants;
- Identification of novel allergens, and toxins, including endogenous and exogenous substances introduced during manufacture;
- Measuring compositional parameters and comparing to conventional products;
- Collecting information on natural variation in the composition of CM and conventional meat products.

*Research needed to develop methods and safety approaches*

- Adapting and validating modern microbial assessment methods including sequencing and -omics approaches;
- Identifying potential interferents in testing (e.g., media, etc.);
- Designing GMP and HACCP approaches suited for CM;
- Establishing safe limits for maximum passage;
- Developing and validating methods to evaluate genetic drift;
- Developing approaches to residue risk assessment;
- Expanding the TTC to include classes of substances relevant to CM (i.e., bioactive molecules);
- Developing methods to identify novel toxins or allergens, including approaches to compare to conventional products;
- Evaluating the stability of inputs and metabolites;
- Determining relevant parameters to characterize cell lines;
- Establishing criteria for conventional comparators and identifying compositional parameters that support safety evaluation or nutritional assessment;
- Developing relevant comparative assessment approaches and acceptable ranges of nutritional and compositional parameters.

*Making data and methods publicly available*

- Establishing publicly available parameter databases for composition, common inputs, and microbiological parameters;
- Publishing peer-reviewed safety research in the public domain;
- Developing standard safety testing methods that provide industry-wide parameters.

## 6. Next Steps

Multistakeholder networks are envisioned as the next step, to develop safety testing methods, establish databases and data sharing mechanisms, and advise or set standards for the field. Collaborative efforts to advance key areas of knowledge around CM create public sources of information and data to address important questions for market and regulatory acceptance. Efforts are needed to broaden engagement across stakeholder groups, reaching academic scientists, consumer organizations, CM developers, established food manufacturers, supporting industries (e.g., suppliers, food safety experts, analytical labs, distributors), regulators, policymakers, and more.

The research priorities identified in this project require this type of multistakeholder effort. Several phases of work are needed, starting with consideration of how diverse stakeholders can meaningfully participate across diverse sectors, disciplines, and geographies in research aiming to develop open-access assessment tools and databases helpful for risk assessment, safety evaluations, and creating consumer confidence in CM products. Establishment of a consortium based on a public/private partnership model (a multistakeholder collaborative group managed by a coordinating organization with both non-profit and for-profit organizations), where task-oriented committees convene experts on specific topical areas for analytical methods development and coordinate research and report findings publicly could be valuable to efficiently address the research priorities. Some examples of successful models include the NanoRelease project [102], the NSF funded Industry-University Cooperative Research Centers Program [103], and Infogest [104].

Overall, the participants in both Phase 1 and Phase 2 of the CMSI initiative were eager to engage in coordinated multistakeholder efforts to advance safety evaluation, and agreed that such efforts would accelerate safety assessment, make evaluations more consistent and reliable, and improve consumer confidence in CM products.

## Figures and Tables

**Table 1:** Workshop discussion topics.

Discussion Topic	Key Discussion Points
Approaches to compositional analyses for safety assessment of cultured meat and seafood	How to define “similarity”? What are important parameters? What are appropriate comparators? What is relevant for safety assessment? (See Issue Paper for full list of discussion points)
Risk assessment of inputs (culture media, components, specifications)	Should residue testing be conducted on all components? Are there common inputs? Could there be generic media or other components that are standardized and shown to be safe? Is there a way to standardize “food grade” requirements for culture media? What methods are appropriate? (See Issue Paper for full list of discussion points)
Microbial and chemical hazards for consumers	How to identify and mitigate microbiological or chemical hazards in final products. What is different from conventional meat production and industries with similar processing machinery? When and how should products be tested? Are current testing methods valid? (See Issue Paper for full list of discussion points)

**Table 2:** Analysis of topics raised in interviews.

Theme	Frequency	Examples of Participant Comments
Process inputs, media, nutrients, matrices	8	“Characterization of hazards starts with inputs; what are the safety of the inputs, steps and processing, which are the possible hazards that could be introduced or removed along the way?” “But the puzzling piece is understanding all the variety of inputs—there are so many inputs that could go in… there could be non-edible components, media used for growth, other types of inputs—looking at what they might be, a more comprehensive understanding would be helpful.” “What’s new? We need to figure out how well characterized the inputs are.” “We need to consider when the inputs occur in the system, too.”
Standards of Identity/Specifications/Nutritional Profile	7	“Understanding the nutritional profile of the product is important; if just cells from muscle, could be different than traditional meat. What they’ve seen in literature, seen researchers adding different components (e.g., b-carotene, alpha-gal negative products). Will be interesting to see how the final products end up and how they compare to traditional meat products.” “In terms of safety, it will come down to: (in terms of base protein material, notwithstanding additional constituents added), composition [of] chemical equivalents to conventional counterpart—for example, for chicken grown safely in cell culture, does it have all the same nutrient profiles (e.g., amino acid)? That provides a good starting point for safety.” “How similar is similar enough?” “Figuring out the metrics, and the comparator can be a challenge.” “We know there will be changes to the cells. So, it’s a matter of finding out what the changes are, and whether they are important for food safety.” “[Demonstrating] similarity may be conceptually similar to the assessment of GM food, e.g., [does] chicken meat [have] the same macro/micronutrient profile as conventionally raised, within natural variation. This may be a compelling argument in terms of safety and nutritional adequacy.”
Microbiological hazards	6	“We are hearing …potential for microbiological contamination relative to conventional meat products is much less. But until we see the component inputs and the kind of conditions they are being made, it’s difficult to predict.” “In general, the manifestation of the [microbial] hazards may be slightly different, but conceptually they are all similar to issues that are already considered during traditional analysis. Just how they present themselves may be slightly different.”
Convergence in technology and processes	4	“The inputs may be different, but if the method of production—e.g., fermenters—could be standardized, perhaps a regulator could say it was done according to Standard XYZ, and wouldn’t have to look into it further.” “If there are standardized inputs—e.g., basal media that was standardized … from a pre-market perspective, would narrow down the needs from pre-market submission.”
Framework/ approaches for analyzing/ interpreting data	4	“There may be issues using some methods that are not well published or validated e.g., for cell cultured components.” “Is there scope for using -omics technology?...In more modern assessments, companies aren’t using these yet to support the safety—so are they even ready to be utilized for cultured meat and seafood? If they are not, what other methods are sufficient to give a reasonable level of assurance?” “A lot of companies are able to provide sequencing data these days because they would use it for their own purposes anyway. From a regulatory/safety standpoint—it would be good to understand how that data can be used. If regulators are going to ask for this, we need to understand how to interpret this data.”
How to assess Protein quality/ Nutritional quality	4	“How do you show the protein is exactly the same?... What is the proper test to show that the protein quality, even the make-up of the fat that is added—how can you show it’s similar to what’s found in traditional meat?” “Impacts on nutrition [are] important—what are these products providing more (or less) than normal meat products… [for example], low protein content compared to conventional meat?” “Also consider nutritional equivalence—what is the purpose of the products? Are they like-by-like replacements, or niche products that aren’t widely consumed?“ “Companies may try to intentionally manipulate nutritional profiles, but [these] may also be unintentional.”
Genetic drift	3	“Genetic drift and adaptation during culture [is] something discussed a lot, and often brought up a lot by people who are in the therapeutic space.” “But for immortal cells I can’t imagine what needs to be demonstrated. Phenotypic? Genetic level? Maybe at a cytogenetic level…” “[One] issue is about genetic drift…When we think about genetic drift information, a useful one is to use sequencing data. How much useful information can be gained from it? That’s debatable. There are some companies that go another way and observe the physical characteristics as a proxy for safety but on the other hand, if you do it that way, is it enough?”
Immortalization and continual cell proliferation and relationship to tumorigenicity	3	“In tissue culture, cells are not cancerous (in the sense that they do not cause cancer to spread to other cells as a carcinogenic chemical inducer or promoter might), so [we] don’t see how eating them would cause a risk, but on the other hand, I can’t say I could convince any friend of mine that they don’t have to be concerned about that. So not sure this is a compelling answer… you might want to come up with a theoretical rationale for considering it one way or another… [and] tests that resolve any scientific questions.” “One thing that will be a challenge is risk communication—[for example,] concerns about uncontrolled cell proliferation and its relationship conceptually to tumorigenicity. This is something that is repeatedly flagged as a risk perception issue.”

## Data Availability

Data is contained within the article.

## Supplementary Materials

The following supporting information can be downloaded at: https://www.mdpi.com/article/10.3390/foods12142645/s1, List of Participant Organizations (Supplementary Materials S1) [105,106,107,108,109,110,111,112,113,114,115], Issue Papers (Supplementary Materials S2).
