# Supplementary material for: Cultured Meat Safety Research Priorities: Regulatory and Governmental Perspectives
Source: Foods. 2023 Jul 8;12(14):2645. doi: 10.3390/foods12142645 (PMC10379195; doi:10.3390/foods12142645)
Supplement: Supplementary file 1 [file foods-12-02645-s001.zip › foods-2374563-supplementary-1.pdf]

New Harvest Cultured Meat Safety Initiative (CMSI)

Overview of topics of interest identified in interviews with regulatory experts

Background

Cultured meat and seafood product development may soon outpace capacity for regulatory evaluation and thereby create bottlenecks at the final stages of product development. In light of anticipated bottlenecks, CMSI's goal is to create a more predictable innovation environment by fostering research on and development of safety assessment methods, data sets and standard practices for key pre-competitive components of product development. This paper reviews the full range of topics raised during CMSI interviews with governmental and regulatory experts as a basis to identify priorities for research and development of those methods, data sets and standards. A summary of all topics is presented here, accompanied by three Issue Papers developed as background for discussion of select research needs and priorities, identified by the team based on the most frequently raised topics during interviews, for the upcoming workshop:

- Issue Paper 1 addresses approaches to compare cultured meat and seafood products to conventional products, and the suitability of comparative analyses to perform food safety assessments;
- Issue Paper 2 focuses on potential hazards associated with the inputs used to manufacture cultured meat and seafood, the approaches required to evaluate risk, and the specifications related to the inputs; and
- Issue Paper 3 focuses on the methods used to assess contamination by microbiological and chemical hazards.

Methods

The central task of CMSI is to identify and prioritize research, methods and standards development to address issues most likely to be encountered during safety assessment of finished cultured meat and seafood products. CMSI Phase 1 worked with innovators to provide a framework and assess industry views of potential areas of focus for questions about safety assessment of CM (Ong et al. 2021). CMSI Phase 2 was initiated with interviews of experts in 8 international regulatory agencies to identify potential topics of interest to support cultured meat and seafood safety assessment. A scripted interview set was used in video conference with experts at regulatory agencies in multiple jurisdictions. Notes taken during the interviews were evaluated for mention of topics of uncertainty with regard to evaluation of research priorities. All interviews and workshops are held under the Chatham House Rule; data are anonymized and aggregated, and identities and affiliations of specific comments will not be publicly revealed.

Key topics were identified based on one-on-one interviews with regulatory scientists. Topics were prioritized based on the number of times that a topic was mentioned or the duration of discussion that the topic received across interviewees. The top three topics will be the initial

focus for discussion at the workshops; however, discussion of the other topics will be encouraged at the workshops.

Regulatory and governmental scientists have been invited to attend either an in-person workshop held on Oct 31, 2022 in Singapore, or a virtual workshop in mid-November. Workshop summaries will be shared as open-source information with all participants for further input. We will use the knowledge gained through the discussions to develop findings to be shared in open source peer-reviewed publications.

Outcomes

Very few agencies have experience in processing full applications for cultured meat and seafood products and consequently the responses to interview questions were often of the nature of, "we will respond to what we see in the data sets submitted" and "there are too many variables across product types to make general statements of hazard or risk." However, most respondents also stated that methods were available to evaluate safety of CM products but that substantial challenges are likely to be encountered by manufacturers in developing sufficient data to apply the methods. Respondents also indicated that the variety of CM product types and processing approaches meant that many of the hazards were related to specific potential issues that would not apply to all cultured meat and seafood products. Therefore, the list of topics for research that CMSI presents in this review should not be viewed as a list of hazards that regulatory experts see for *all* or even for *any* individual CM product.

The initial categorization of topics is presented in Table S1a (selected as top 3 topics), and S1b (other potential discussion topics). Selection of questions and topic categories by CMSI should be viewed as an initial sorting to support discussion among regulatory and governmental experts of how to progress toward identification of research priorities. Re-statement of issues by experts is encouraged at the workshops, as is re-sorting of the priority order, or identification of topics not listed here. Similar topics were combined to develop Issue Papers that address the top 3 priority topics.

Table S1a – Priority topics for discussion at the workshops

**Issue paper**	**Topic**	**Key discussion points**
Issue paper 1	Approaches to compositional analyses for safety assessment of cultured meat and seafood	How to define ‘similarity’? What are important parameters. What are appropriate comparators? What is relevant for safety assessment?<br>(see Issue Paper for full list of discussion points)

Issue paper	Topic	Key discussion points
Issue paper 2	Risk assessment of inputs (culture media, components, specifications)	Should residue testing be conducted on all components? Are there common inputs? Could there be generic media or other components that are standardized and shown to be safe? Is there a way to standardize 'food grade' requirements for culture media? What methods are appropriate?<br><br>(see Issue Paper for full list of discussion points)
Issue paper 3	Microbiological & chemical hazards for consumers	How to identify and mitigate microbiological or chemical hazards in final products. What is different from conventional meat production and industries with similar processing machinery? When and how should products be tested? Are current testing methods valid?<br><br>(see Issue Paper for full list of discussion points)

Table S1b: Other topics identified as key issues

Topic	Discussion points
Convergence in technology and processes	Where might convergence and potential for standardizing or streamlining aid assessment in precompetitive space? (*e.g.*, for manufacturing processes, bioreactors, basal media)
Framework and approaches for analyzing or interpreting data	How can datasets useful for characterizing outputs of cell culture and complex mixtures (e.g., *in vitro* assay arrays, -omics) be analyzed in a meaningful way (*e.g.*, grouping/read across, tiered assessment)?
Guidance for preventing contamination	What monitoring practices are needed for place/timing considering differences in technologies and processing steps (*e.g.*, considering master cell banks, working cell banks and source cells). Consider similarities and differences with conventional meat processing and fermentation and development of food safety plans.
Genetic drift	How would the possibility of genetic drift in cell lines be considered and addressed (*e.g.*, with respect to GMP generally or introduction of variation in protein structures)?

Topic	Discussion points
Assessment of protein quality/nutritional quality	How to monitor changes to dietary protein derived from cell lines rather than whole animal (*e.g.*, tertiary structure variation) or isolated proteins with respect to nutritional comparison and health outcome evaluation (*e.g.*, allergens).
Immortalization cell proliferation relationship to tumorigenicity	Does this pose a risk, and if so, how to assess?
Characterization of cells	Cells may come from any species and cell type. Techniques used to generate cellular components (immortalized cells, genetic modification) will also vary. What methods/approaches are needed to characterize consistently and aid regulatory and safety evaluation?

Goals of the workshop

The overall goal is to develop a comprehensive research strategy for safety of cultured meat and seafood products that reflects the views of regulators to share with diverse stakeholders. The workshop will consider whether data sets, methods or standards development efforts can be developed to address potential safety questions associated with cultured meat and seafood.

CMSI Phase 2 will address these subtasks through workshops and further input from experts at regulatory agencies. As the topics are discussed, participants should consider what types of research and data would make the risk assessment and hazard identification and mitigation process more efficient while supporting the manufacturing of safer products.

New Harvest Cultured Meat Safety Initiative - Issue Paper 1

Approaches to compositional analyses for safety assessment of cultured meat and seafood

The New Harvest/Vireo Cultured Meat Safety Initiative (CMSI) is aiming to identify, prioritize and address research priorities to reduce uncertainties and questions about safety from diverse stakeholder and regional perspectives. Phase 1 addressed industrial and commercial priorities, while the current Phase 2 is gaining input from regulatory and governmental perspectives. This is the first of three issue papers summarizing select topics and inputs from interviews with governmental representatives as background for discussion during the 2022 workshops. This paper focuses on potential approaches that can be used to compare cultured meat and seafood products to conventional products, and the suitability of comparative analyses to perform food safety assessments.

Background

Cultured meat and seafood products are currently derived from conventional meat, poultry, and seafood sources, where the cells are grown in conditions that provide the basic building blocks such as amino acids, vitamins, and minerals needed for tissue development. Therefore, the basic macro- and micro-nutrients of cultured meat and seafood products are likely to be similar to conventional meats, and any differences may be used as a basis to assess for potential food safety concerns. There might be intended modifications or alterations in essential nutrients, including beneficial bioactive chemicals, or compounds that provide better nutrition profile or sensory qualities. For example, addition of myoglobin for increased proliferation of cells and similar coloration to traditional meat; engineering primary bovine cells to produce the antioxidant carotenoids phytoene, lycopene, and β-carotene for increased nutritional value; and engineering scaffolds with striated textures to promote the growth of cells in a structure similar to traditional meat (Simsa et al. 2019, Stout et al. 2020, Seah et al. 2021).

The assessment of novel foods often includes a compositional assessment that is compared to a wild-type or conventional comparator. Novel substances that are compositionally similar to these comparators may be assumed to have a similar safety profile. Where there are differences then there is a need for further assessment.

Measuring the biologically relevant changes in composition, such as change in proximate, vitamin, mineral, amino acid, or fatty acid composition can support assessment of potential toxicological or nutritional concerns. In addition, understanding the similarity to conventional products may help develop dietary exposure evaluation, using data on existing intakes of products of similar nutritional composition.

There is a need to consider how differences may adversely affect:

- Excessive intakes of nutrients or other bioactive substances as a result of unusually high levels in cultured meat or seafood products;
- Substitution for foods of significant nutritive value with less nutritious cultured meat or seafoods; or,
- New or increased levels of toxins, anti-nutrients, or allergens.

There is little public literature describing or evaluating the composition of cultured meat, likely due to a lack of samples available for scientific study and protection of intellectual property. Existing studies indicate that there may be some differences in composition. In one study, the amino acid composition of cultured chicken and cattle meat was assessed to evaluate the effects on the taste profile as compared to conventional meat (Joo et al. 2022). Cultured meat amino acids differed from conventional meat for all but valine and tyrosine as well as glutamic acid for cattle meat (Joo et al. 2022). The use of a peanut wire-drawing protein scaffold for culturing porcine smooth muscle cells resulted in meat that had comparable protein content to traditional meat, but higher moisture and ash content as well as lower fat content (Zheng et al. 2022). These studies highlight that there may be some differences between conventional and cultured meat and seafood products in composition; it is unknown whether other parameters, such as vitamins or mineral content, may also differ in commercial products. No studies are publicly available that have attempted to evaluate these differences in the context of a food safety risk assessment.

Variation in the nutrient composition of conventional meat can be influenced by genetics, along with different feeding and rearing strategies (Juárez et al., 2021). Therefore, there is already a range of ‘natural’ variation in vitamins, trace elements, total protein and amino acids, as well as total fat and fatty acid composition that exists in conventional meats (Juárez et al., 2021). It is anticipated that there will also be a range of nutrient composition among cultured meat and seafood products.

Uncertainties/Questions raised during interviews

In the one-on-one interviews, a number of questions were raised related to the assessment of compositional similarity between cultured meats and conventional products. These include:

- How do we define ‘similarity’? *i.e.,* if a cultured meat or seafood product is highly ‘similar’ to a conventional product, then can the long history of consumption of the conventional meat be used to rely on safety?

- Where is the threshold of ‘different’? How do we define this? Do we use ‘natural’ variation in conventional meats to set upper and lower limits, or is there a percentage tolerance level to allow for exceedance/deficiency of natural ranges? Is there a possibility of reaching quantitative guidance?

- What compositional parameters are important for safety assessment? Some suggestions are: proximate (protein, fat, moisture, ash, carbohydrates), amino acids, vitamins, minerals, fatty acids?

- How do compositional differences relate to food safety? What differences are 'acceptable' from a food safety perspective?

- Is it possible to develop a standard set of parameters to screen for nutritional adequacy and compositional equivalence?

- Is there a need for compositional parameters to be included in 'specifications' or 'standards of identity' for the various categories of cultured meat?

- What are appropriate comparators? What if there is no good comparator (e.g., biopsy from exotic animals, where 'conventional meat' is not available)?

- Does it make sense to do compositional testing of different foods as baseline references and/or for cultured meat as an industry? What is the 'natural variation'?

- Are the existing standard approaches to composition measurement of meat appropriate for cultured meat?

- Given the sheer number and complexity of chemicals in a food that is based on cell growth, it would be impractical if not impossible to identify, isolate and test all the chemical constituents of cultured meat products individually. But, testing of whole cultured meat products in animal models is unlikely to provide the kind of statistical power needed to reach regulatory definitions of safety. Are there appropriate tests for whole foods using new and alternative methods (in vitro, -omics, in silico)?

References

Joo, S.T., Choi, J.S., Hur, S.J., Kim, G.D., Kim, C.J., Lee, E.Y., Bakhsh, A. and Hwang, Y.H., 2022. A Comparative Study on the Taste Characteristics of Satellite Cell Cultured Meat Derived from Chicken and Cattle Muscles. Food Science of Animal Resources, 42(1), p.175.

Juárez, M., Lam, S., Bohrer, B.M., Dugan, M.E., Vahmani, P., Aalhus, J., Juárez, A., López-Campos, O., Prieto, N. and Segura, J., 2021. Enhancing the nutritional value of red meat through genetic and feeding strategies. *Foods*, *10*(4), p.872.

Seah, J.S.H., Singh, S., Tan, L.P. and Choudhury, D., 2022. Scaffolds for the manufacture of cultured meat. *Critical Reviews in Biotechnology*, *42*(2), pp.311-323.

Simsa, R., Yuen, J., Stout, A., Rubio, N., Fogelstrand, P. and Kaplan, D.L., 2019. Extracellular heme proteins influence bovine myosatellite cell proliferation and the color of cell-based meat. *Foods*, *8*(10), p.521

Stout, A.J., Mirliani, A.B., Soule-Albridge, E.L., Cohen, J.M. and Kaplan, D.L., 2020. Engineering carotenoid production in mammalian cells for nutritionally enhanced cell-cultured foods. *Metabolic engineering*, *62*, pp.126-137.

Zheng, Y.Y., Chen, Y., Zhu, H.Z., Li, C.B., Song, W.J., Ding, S.J. and Zhou, G.H., 2022. Production of cultured meat by culturing porcine smooth muscle cells in vitro with food grade peanut wire-drawing protein scaffold. Food Research International, 159, p.111561.

New Harvest Cultured Meat Safety Initiative - Issue Paper 2

Risk assessment of inputs

The New Harvest/Vireo Cultured Meat Safety Initiative (CMSI) is aiming to identify, prioritize and address research priorities to reduce uncertainties and questions about safety from diverse stakeholder and regional perspectives. Phase 1 addressed industrial and commercial priorities, while the current Phase 2 is gaining input from regulatory and governmental perspectives. This is the second of three issue papers summarizing select topics and inputs from interviews with governmental representatives as background for discussion during the 2022 workshops. This paper focuses on potential hazards associated with the inputs used to manufacture cultured meat and seafood, including culture media, components, specification, the approaches required to evaluate safety, and the specifications related to the inputs.

Background

Many inputs such as media, scaffold or microcarriers, antibiotics, and cryoprotectants are required to manufacture cultured meat. Cultured meat begins with a foundation of cells obtained from a biopsy of a desired animal or an immortal cell line from a specific species. To promote the growth, proliferation, and differentiation of cells *in vitro*, they need to be cultured in media, which is a formulation derived from the functional molecules necessary for life. Essential media components include: (1) amino acids added as free amino acids or as hydrolysates, (2) vitamins and minerals in the form of inorganic salts; and (3) carbohydrates, which support the growth of cells and could provide a similar nutritional profile of cultured meat to traditional meat. For cultured meat and seafood, media will also include molecules such as growth factors, which are biologically active molecules capable of stimulating cell proliferation, repair, and differentiation. Different kinds of growth factors have specific roles in the development processes of organisms and may be obtained from animal-derived isolations, plant extracts, microbial fermentation, or other genetically engineered organisms. While there is little information disclosed about what types of media will be commercially used to manufacture cultured meat, public literature suggests that media already used to culture cells for research of pharmaceutical use may be used, such as Dulbecco's Modified Eagle's Medium (DMEM) (O'Neill et al. 2022, Park et al. 2021, Zheng et al. 2022). Other common culture media include Ham's F10, Ham's F12, MEM, Media 199, RPMI 1640, and IMDM (Lee et al. 2022b, Lee et al. 2022a). One group has developed a food-grade version of DMEM, which was made completely from ingredients that have previously been consumed as food or food additives (Kanayama et al. 2022). Culture media is also commonly accompanied by fetal bovine serum (FBS), which is obtained from the blood of fetal calves and contains water, glucose, electrolytes, antibodies, antigens, hormones, growth factors, and more, which supports the proliferation and differentiation of cells. The use of FBS is being either reduced or replaced because: (1) it is derived from an animal source and is a possible source of contamination; (2) it contains

undefined substances; and (3) it is ethically immoral, and expensive (Zhang et al. 2021). Research is being conducted to replace FBS with more sustainable options by creating serum-free media derived from bacterial extracts, algae, fungi, or yeast cells or other supplements such as growth factors, sugars, proteins or chemical additives (Zhang et al. 2021, Lee et al. 2022a).

The process of culturing meat also requires the use of other inputs, such as scaffolds, antibiotics, and cryoprotectants. Scaffolds are artificially engineered structures that are able to support three-dimensional cell growth and tissue formation. They can be made of synthetic, natural, or composite materials which are produced through processes such as recombinant technology, fermentation, extrusion, and bioprinting. Antibiotics kill or inhibit the growth and replication of microorganisms; they are used to prevent or minimize bacterial contamination of cultured cells. Cultured meat producers have indicated that they are avoiding antibiotic use altogether by working in sterile and controlled conditions or only using antibiotics in early stages of production. Cryoprotectants are used during cell storage in low temperatures (such as liquid nitrogen) to protect the cells from damage caused from freezing. To date there has been no publicly available information performing a quantitative food safety risk assessment of inputs, including an evaluation of their potential to be present in the final product.

Guidelines for assessing the safety of the media inputs and the final product have not been firmly established and are still in development by regulatory agencies. The most recent approach to assessing the media inputs of cultured meat was released by the Singapore Food Agency in September 2022. In their guidance, they suggested that the safety of media could be assessed through measuring residual levels of inputs in the final product, using available health-based guidance, available toxicological data, representation of components in the Threshold of Toxicological Concern database, safety guidance for recombinant DNA technology, and the occurrence of the component in identical form in food with a history of safe use. If the media component does not have a history of safe use in food, then it is necessary to evaluate the safety concerns surrounding the substance such as allergenicity, potential presence of viruses or prions, and other toxicity concerns. The decision tree to assess the safety of biological media substances is shown in Figure S1.

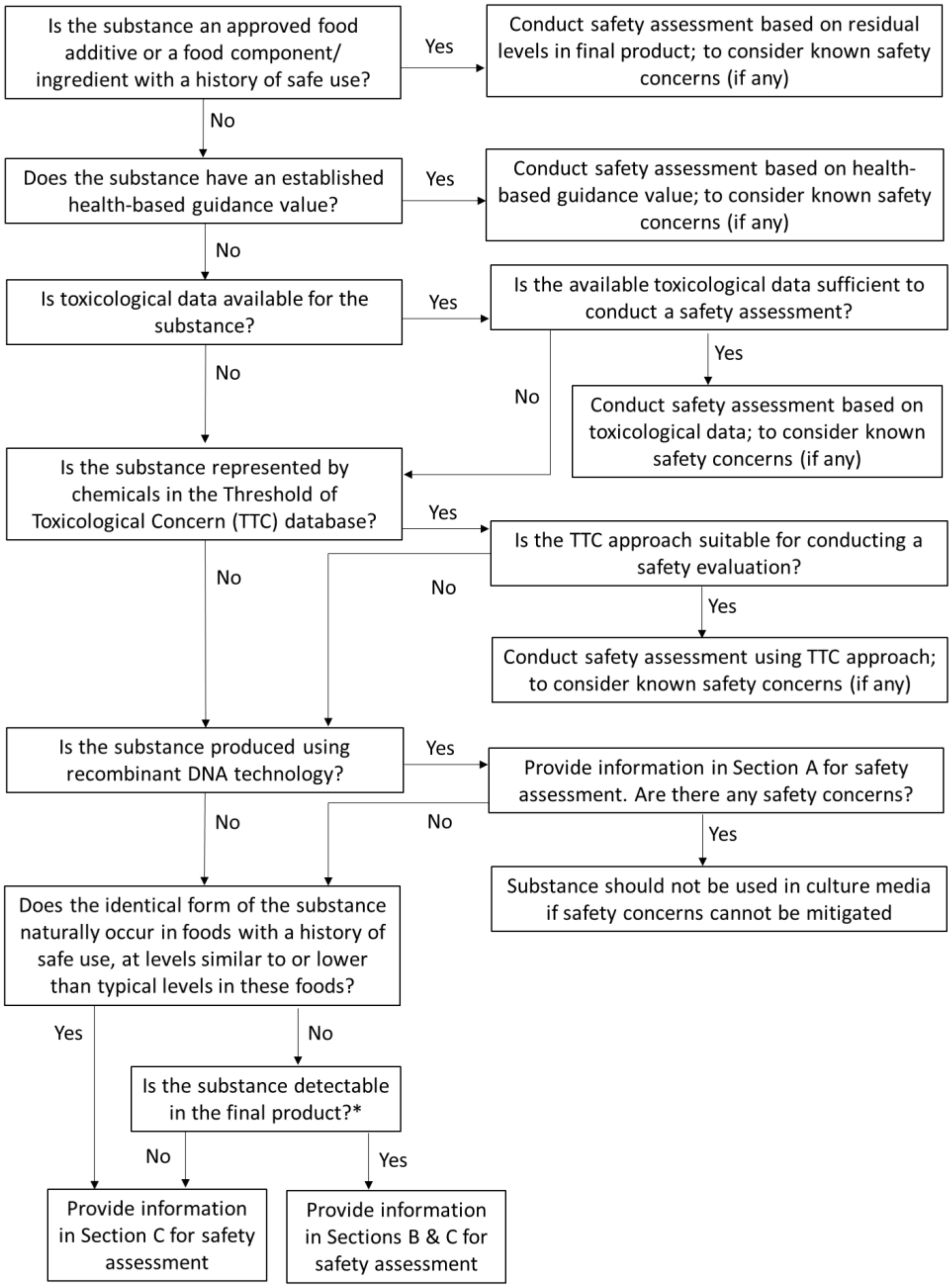

**The detection methodology and limit of detection must be comparable to scientifically established detection methodologies for the substance*

Figure S1. Singapore Food Agency Decision Tree (Sept 2022) Safety assessment approach for biological substances used in media for cultured meat/seafood production

There may be a need for new evaluation procedures to assess the quality, safety and purity of culture media components and other inputs. Experts suggested that the tools and assessment methods currently available to measure and mitigate potential hazards may be adequate for

case-by-case assessment of cultured meat and seafood products. For example, residue analysis, acceptable intake determination for specific constituents, and evaluation of specific endpoints such as allergenicity.

Currently, food ingredients and the equipment used to manufacture and prepare food is suggested to be “food grade”. Generally, food grade quality ingredients are assumed to meet the standards of safety for human consumption, while food grade equipment/contact substances do not contain toxic components which could come into contact or leach into food.

However, more concrete approaches to certifying an input as “pharmaceutical-grade” exist. For a material to be considered pharmaceutical grade, products must meet certain purity standards (often >99% with no extra binders or fillers), be produced under pharma-Good Manufacturing Practices (GMP), and meet or exceed requirements of an established pharmacopeial standard (*e.g.,* United States, European, and British Pharmacopoeia). Raw materials used to make pharmaceutical grade substances also need to be considered pharmaceutical grade. The facilities used to manufacture pharmaceutical grade substances are held to GMP ensuring that facilities and workers are sanitary, production is uniform between batches, and that testing is performed to confirm the activity and sterility of the produced drug in random samples. Pharmaceutical-grade materials such as media are cost-prohibitive for use in large-scale cultured meat and seafood production. Other grades exist, such as reagent grade (meets or exceeds American Chemical Society standards and >95% purity) or laboratory and technical grade (not well defined, but intended for research, commercial, or industrial purposes). However, these grades of inputs have not been qualified for use in food.

Therefore, the approach to qualifying media and other inputs as ‘food-grade’ and appropriate for use in cultured meat and seafood needs to be developed.

Uncertainties/ Questions raised during interviews

In the one-on-one interviews, a number of questions were raised related to the assessment of inputs. These include:

- How much of the media components, scaffold, antibiotics, cryoprotectants, and other inputs are present in the final products?
- Should residual testing be conducted on the media components used in production? And if so, does testing need to be conducted on all the components used or only certain substances?
- Will media need to be approved on a case-by-case basis, or will there be regulations/restrictions on the components which can be used in culture media?
- Are there inputs that are commonly used across all processes? Could there be a generic culture media product that is shown to be safe?
- Is there a way to standardize ‘food-grade’ for cultured meat and seafood inputs

- What quantitative or qualitative approaches are appropriate to assess media components?
- What methods are appropriate for testing?
- Should specifications for the final product include any residues? Are specifications going to be established on a case-by-case basis?
- Are current food additive specifications (e.g., Joint FAO/WHO Expert Committee on Food Additives) applicable for cultured meat and seafood?
- What level of impurity is acceptable in the inputs?
- How should genetically engineered inputs be evaluated?
- Are current antimicrobial limits established for meat appropriate for cultured meat and seafood?
- Is there a potential risk if the input is present in the final product at lower or equivalent levels than in conventional foods?
- How should processing aids be defined and evaluated in the context of cultured meat and seafood? Are there different safety considerations between an additive and a processing aid?
- How should growth factors be assessed?

References

Kanayama, T., Tanaka, K., Shimada, H., Ichiyama, K., Higuchi, S., & Kawashima, I. (2022). Improving the Safety of Cultured Meat Using Basal Medium Prepared using Food Ingredients. https://doi.org/10.1101/2022.03.28.486069

Lee, D. Y., Lee, S. Y., Won Jung, J., Hyun Kim, J., Hun Oh, D., Woo Kim, H., Hyeop Kang, J., Seok Choi, J., Kim, G.-D., Joo, S.-T., Jin Hur, S., won Jung, J., & woo Kim, H. (2022). Review of technology and materials for the development of cultured meat. Https://Doi-Org.Wv-o-Ursus-Proxy02.Ursus.Maine.Edu/10.1080/10408398.2022.2063249, 1–25. https://doi.org/10.1080/10408398.2022.2063249

Lee, D. Y., Lee, S. Y., Yun, S. H., Jeong, J. W., Kim, J. H., Kim, W. H., Choi, J. S., Kim, G.-D., Joo, S. T., Choi, I. H., & Hur, S. J. (2022). Review of the Current Research on Fetal Bovine Serum and the Development of Cultured Meat. Food Science of Animal Resources, 42(5), 775–799. https://doi.org/10.5851/KOSFA.2022.E46

O'Neill, E. N., Ansel, J. C., Kwong, G. A., Plastino, M. E., Nelson, J., Baar, K., & Block, D. E. (2022). Spent media analysis suggests cultivated meat media will require species and cell type optimization. Npj Science of Food 2022 6:1, 6(1), 1–10. https://doi.org/10.1038/S41538-022-00157-Z

Park, S., Jung, S., Choi, M., Lee, M., Choi, B., Koh, W. G., Lee, S., & Hong, J. (2021). Gelatin MAGIC powder as nutrient-delivering 3D spacer for growing cell sheets into cost-effective

cultured meat. Biomaterials, 278, 121155. https://doi.org/10.1016/J.BIOMATERIALS.2021.121155

Singapore Food Agency. Sept 2022. Requirements for the Safety Assessment of Novel Foods and Novel Food Ingredients. https://www.sfa.gov.sg/docs/default-source/food-import-and-export/Requirements-on-safety-assessment-of-novel-foods_26Sep.pdf

Zhang, L., Hu, Y., Badar, I. H., Xia, X., Kong, B., & Chen, Q. (2021). Prospects of artificial meat: Opportunities and challenges around consumer acceptance. Trends in Food Science & Technology, 116, 434–444. https://doi.org/10.1016/J.TIFS.2021.07.010

Zheng, Y. Y., Chen, Y., Zhu, H. Z., Li, C. B., Song, W. J., Ding, S. J., & Zhou, G. H. (2022). Production of cultured meat by culturing porcine smooth muscle cells in vitro with food grade peanut wire-drawing protein scaffold. Food Research International, 159, 111561. https://doi.org/10.1016/J.FOODRES.2022.111561

New Harvest Cultured Meat Safety Initiative - Issue Paper 3

Microbiological & Chemical Hazards for Consumers

The New Harvest/Vireo Cultured Meat Safety Initiative (CMSI) is aiming to identify, prioritize and address research priorities to reduce uncertainties and questions about safety from diverse stakeholder and regional perspectives. Phase 1 addressed industrial and commercial priorities, while the current Phase 2 is gaining input from regulatory and governmental perspectives. This is the third of three issue papers summarizing select topics and inputs from interviews with governmental representatives as background for discussion during the 2022 workshops. This paper focuses on the methods used to assess contamination by microbiological and chemical hazards.

Background

Because it is produced in contained conditions, cultured meat may present different microbial safety profiles as well as resistance to deterioration and spoilage compared to traditional livestock production. However, microbial contamination with adventitious agents such as bacteria, fungi, viruses, parasites, and prions can occur throughout the entire manufacturing process and can be considered in three broad categories:

- Contamination introduced with the tissues of source animals;
- Contamination introduced with other manufacturing inputs such as cell media components and reagents;
- Contamination introduced from the production environment.

Meanwhile, chemical contamination can originate from water sources (Cobo et al., 2005; Bhagwat, V. R., 2019) or from inorganic and organic leachable substances and chemicals such as plasticizers from disposable products (*e.g.*, cell culture plastics, filters), coatings on equipment, packaging materials, and cleaning products. Equipment and disposables used in cultured meat and seafood manufacture may not be specially designed to be used in food, or previously have been used for food.

Cultured meat and seafood products may challenge existing methods to measure or mitigate potential health risks derived from microbiological and chemical hazards. However, the challenges expressed by regulatory and governmental experts were almost uniformly related to complexity of analysis rather than technical feasibility.

Monitoring and assessment methods may be adapted from conventional meat and seafood processing, although the techniques may not be entirely applicable. The sampling approach (locations and frequency, and the types of microbial species tested will likely differ for cultured meat and seafood. Microbial contamination in meat from slaughtered animals is often related to bacteria that reside in animals, in the digestive tract, and in feces (Rhoades et al., 2009). But there is currently little publicly available data on the specific microbial and chemical contaminants that may be introduced during cultured meat production. Cultured meat and seafood production takes place in sterile conditions and does not require prolonged handling of

live animals. Products are less susceptible to contamination from these sources. However, if contamination from microbes such as *E. coli* occurs during the procurement of tissues or cells from animals, it would likely rapidly outgrow the cell culture and would result in disposal of that batch.

Safety assurance methods for cell culture processes used to produce food grade enzymes and medical biologics may also apply to cultured meat and seafood products (Bal-Price and Coecke, 2011). Biologics or cells intended for pharmaceutical or clinical use undergo extensive testing for pathogens. However, many of the pathogens that are of concern in those products are only hazards if introduced through direct injection or as part of tissue or cell implantation, where the cells are maintained in the viable state. Cells are not expected to remain viable in cultured meat and seafood products when presented to consumers. Therefore, it is unknown which microbes have the potential to cause foodborne illnesses in cultured meat or seafood products.

Good Manufacturing Practices (GMP), development of Hazard and Critical Control Point (HACCP) plans, and Good Cell Culture Practice (GCCP) provide well established protocols to evaluate and control microbial and chemical contaminants in a range of current production environments (Bal-Price and Coecke, 2011; Bucknavage and Campbell, 2020; Pampoukis et al., 2022; Sung and Hawkins, 2020). These prescribe aseptic handling for cells and continual monitoring for microbial growth and contamination. They also prescribe approaches to address contamination risks that are common for food production: (1) risks from personnel that may harbor agents related to infectious disease; (2) risks from cross-contamination from allergens such as wheat, dairy, shellfish, and nuts; (3) risks from water that may bring microbiological contamination; and (4) risks from chemical contamination that can leach from equipment, glassware or pipes. These are baseline requirements in many countries to produce food acceptable for commercial sale, and theoretically should be applicable to manage microbial and chemical hazards of cultured meat and seafood products, though an evaluation may be needed to identify relevant elements of GCCP for food production (Ong et al., 2021).

Uncertainties/Questions raised during interviews

In the one-on-one interviews, a number of questions were raised related to the assessment of microbial and chemical contamination in cultured meats and seafoods. These include:

- Research is needed to validate the use of current testing techniques for cultured meat and seafood. Are there approaches to sample collection (*e.g.,* types of sample, location, frequency, types of microbes) that differ from sampling plans and testing in conventional meat processing?
- Compounds used in cultured meat and seafood products may inhibit the use of polymerase chain reaction methods, or change the results of mass spectrometry (*e.g.,* Rossen et al., 1993). Are there matrix interferences that affect microbiological and chemical detection and quantitation limits?
- Can biological or chemical contamination accumulate in bioreactors or other equipment?

- Pathogens not typically associated with meat or seafood may grow in cultured meat products due to the types of growth media and lack of competing microorganisms in cultured meat products. Current monitoring methods may not detect these pathogens (Jiang et al., 2021; Krska et al., 2012; Xia et al., 2022).
- If a final product is sterile, is it easier for harmful microbes to colonize the product (*i.e.,* no competition from surface flora)?
- Are there any possible novel microbial hazards such as viruses that integrate into the genome at various stages of production?
- Is there a potential for the presence of prions and zoonotic pathogens from cell lines derived from atypical food animals or atypical tissues (*e.g.,* brain tissue)?
- Does knowledge of industries with 'similar' processing machinery used for products such as milk, brewery, precision fermentation, allow for bridging or "read across" approaches to comprehend the likelihood of hazards for cultured meat and seafood products?

References

Bal-Price, A., and S. Coecke, 2011, Guidance on Good Cell Culture Practice (GCCP), *in* M. Aschner, C. Suñol, and A. Bal-Price, eds., Cell Culture Techniques: Totowa, NJ, Humana Press, p. 1-25.

Bucknavage, M., and J. A. Campbell, 2020, Good manufacturing practices and other programs in support of the food safety system, Food Safety Engineering, Springer, p. 159-173.

Bhagwat, V. R., 2019, Safety of Water Used in Food Production, Food Safety and Human Health, Elsevier Inc., p. 219-47.

Cobo, F., G. N. Stacey, C. Hunt, C. Cabrera, A. Nieto, R. Montes, J. L. Cortés, P. Catalina, A. Barnie, and Á. Concha, 2005, Microbiological control in stem cell banks: approaches to standardisation: Applied Microbiology and Biotechnology, v. 68, p. 456-466.

Ong, K. J., J. Johnston, I. Datar, V. Sewalt, D. Holmes, and J. A. Shatkin, 2021, Food safety considerations and research priorities for the cultured meat and seafood industry: Comprehensive Reviews in Food Science and Food Safety, v. 20, p. 5421-5448.

Pampoukis, G., A. E. Lytou, A. A. Argyri, E. Z. Panagou, and G.-J. E. Nychas, 2022, Recent Advances and Applications of Rapid Microbial Assessment from a Food Safety Perspective: Sensors, v. 22.

Jiang, L., M. M. Hassan, S. Ali, H. Li, R. Sheng, and Q. Chen, 2021, Evolving trends in SERS-based techniques for food quality and safety: A review: Trends in Food Science & Technology, v. 112, p. 225-240.

Krska et al., 2012: Krska, R., A. Becalski, E. Braekevelt, T. Koerner, X.-L. Cao, R. Dabeka, S. Godefroy, B. Lau, J. Moisey, D. F. K. Rawn, P. M. Scott, Z. Wang, and D. Forsyth, 2012,

Challenges and trends in the determination of selected chemical contaminants and allergens in food: Analytical and Bioanalytical Chemistry, v. 402, p. 139-162.

Rhoades, J. R., G. Duffy, and K. Koutsoumanis, 2009, Prevalence and concentration of verocytotoxigenic Escherichia coli, Salmonella enterica and Listeria monocytogenes in the beef production chain: A review: Food Microbiology, v. 26, p. 357-376

Rossen, L., Nørskov, P., Holmstrøm, K. and Rasmussen, O.F., 1992. Inhibition of PCR by components of food samples, microbial diagnostic assays and DNA-extraction solutions. International journal of food microbiology, 17(1), pp.37-45.

Sung, J., and J. R. Hawkins, 2020, A highly sensitive internally-controlled real-time PCR assay for mycoplasma detection in cell cultures: Biologicals, v. 64, p. 58-72.

Xia et al., 2022: Xia, X., H. Yang, J. Cao, J. Zhang, Q. He, and R. Deng, 2022, Isothermal nucleic acid amplification for food safety analysis: TrAC Trends in Analytical Chemistry, v. 153, p. 116641.
