# Supplementary material for: Cultured Meat Safety Research Priorities: Regulatory and Governmental Perspectives
Source: Foods. 2023 Jul 8;12(14):2645. doi: 10.3390/foods12142645 (PMC10379195; doi:10.3390/foods12142645)
Supplement: Supplementary file 1 [file foods-12-02645-s001.zip › foods-2374563-supplementary-2.pdf]

Supplement S2

List of interview and workshop participant affiliations

European Food Safety Authority

Food Standards Australia New Zealand

Kenya Bureau of Standards

National Food Service, Ministry of Health, Israel

Singapore Food Agency

National University of Singapore (NUS)

Duke-NUS Medical School

US Food and Drug Administration

Zambia National Biosafety Authority

Governmental representatives from 6 additional undisclosed jurisdictions
